# The effect of personality on likelihood of contracting SARS-CoV-2 in the United States

**DOI:** 10.1016/j.paid.2025.113549

**Published:** 2025-11-19

**Authors:** Joshua K. Wood, Sara J. Weston, David M. Condon, Heather Kalish, Carleen Klumpp-Thomas, Holly Ann Baus, Jing Wang, Jennifer Mehalko, Jameson Travers, Kyle Pauly, Jennifer A. Croker, Yan Li, Lindsay Czajkowski, Cheryl Chairez, Kelly Snead, Alison Han, Luca T. Giurgea, Luz Angela Rosas, Rachel Bean, Rani Athota, Adriana Cervantes-Medina, Rocco Caldararo, Michelle M. Kolberg, Andrew Kelly, Reid Simon, Saifullah Shafiq, Susan Reed, Eric W. Ford, Sam G. Michael, Robert P. Kimberly, Steven E. Reis, Dominic Esposito, Matthew J. Memoli

**Affiliations:** a School of Psychology, Deakin University, Geelong, Australia; b Department of Psychology, University of Oregon, Eugene, OR, USA; c Trans-NIH Shared Resource on Biomedical Engineering and Physical Science, National Institute of Biomedical Imaging and Bioengineering, National Institutes of Health, Bethesda, MD, 20894, USA; d National Center for Advancing Translational Sciences, National Institutes of Health, Rockville, MD, 20850, USA; e Clinical Studies Unit, Laboratory of Infectious Diseases, National Institute of Allergy and Infectious Diseases, National Institutes of Health, Bethesda, MD, 20894, USA; f Clinical Monitoring Research Program Directorate, Frederick National Laboratory for Cancer Research, Frederick, MD, 21702, USA; g Protein Expression Laboratory, NCI RAS Initiative, Frederick National Laboratory for Cancer Research, Frederick, MD, 21702, USA; h Center for Clinical and Translational Science, School of Medicine, University of Alabama at Birmingham, Birmingham, AL, 35294, USA; i Joint Program in Survey Methodology, Department of Epidemiology and Biostatistics, University of Maryland College Park, College Park, MD, 20742, USA; j Laboratory of Immunoregulation, National Institute of Allergy and Infectious Diseases, National Institutes of Health, Bethesda, MD, 20894, USA; k Clinical Research Directorate, Frederick National Laboratory for Cancer Research, Leidos Biomedical Research, Inc., Frederick, MD, 21702, USA; l Division of Clinical Research, National Institute of Allergy and Infectious Diseases, National Institutes of Health, Bethesda, MD, 20894, USA; m Clinical and Translational Science Institute, University of Pittsburgh, Pittsburgh, PA, USA

**Keywords:** COVID-19, SARS-CoV-2, Personality, Big Five, Risk attitudes, Health

## Abstract

This study investigated whether personality differences affect the likelihood of contracting SARS-CoV-2, the virus that causes COVID-19. Personality is associated with personally controllable virus protective behaviors, and adoption of these protective measures influences the likelihood of contracting SARS-CoV-2. Previous research also indicates relationships between personality and virus infection. Therefore, it was predicted that personality differences would influence the likelihood of contracting SARS-CoV-2. Participants were 6158 volunteers (53 % female, age *M* = 50.63, *SD* = 15.33) in the United States. Between April 2020 and November 2021, participants had one to three serological tests for antibodies to SARS-CoV-2, approximately 6 months apart, and completed measures of Big Five personality, risk perception, risk taking, and optimism. After controlling for demographic variables, the openness factor had a significant negative association with seropositivity, even with strict corrections for multiple comparisons. However, when also controlling for county COVID prevalence, as pre-registered, predictions of trait-seropositivity associations were not supported. These results suggest that commonly assessed personality traits do not substantially influence the likelihood of contracting SARS-CoV-2. We conclude that situational factors (e.g., capacity to avoid others, COVID prevalence) have a greater influence than personality on the likelihood of infection.

## 1. Introduction

Severe acute respiratory syndrome coronavirus type 2 (SARS-CoV-2) is the virus that causes coronavirus disease 2019 (COVID-19). In response to the COVID-19 pandemic, governments around the world generally recommended or enforced adherence to personally controllable protective behaviors, including: self-isolation from others (shelter-in-place orders), physical or social distancing (e.g., staying 1.5 m from others when in their presence), mask wearing, hand-washing, and eventually vaccinations.

Evidence indicates that personality plays a role in whether individuals adopt behaviors designed to reduce risk of infection (e.g., Nofal et al., 2020). Assuming these behaviors are effective at preventing SARS-CoV-2 infection, personality should have an indirect effect on the likelihood of contracting SARS-CoV-2.

### 1.1. Asymptomatic infection

Asymptomatic infection is infection without perceptible symptoms. In serological testing of a representative sample in the United States, it was estimated that 5 months into the pandemic in 2020 approximately five additional people were infected with SARS-CoV-2 for every person who was officially detected and counted in prevalence figures (Kalish et al., 2021). Reported effects of personality on contraction of the virus have mostly been made on the basis of self-reported infection. Thus, there is a need for research using serological measurement—blood tests of infection, indicated by antibodies to SARS-CoV-2—to take into account the substantial number of undiagnosed SARS-CoV-2 infections.

### 1.2. The influence of personality and demographics on protective behaviors and infection

Studies based on self-reports have evaluated the relations between psychological characteristics and the adoption of protective behaviors. We now summarize prior findings on the influence of personality on protective behaviors. These findings relate directly to our hypotheses, as greater adoption of protective behaviors should be associated with lower risk of infection.

#### 1.2.1. Big Five model of personality

Personality is an individual’s typical pattern of thought, emotion, and behavior, that is comparatively stable over time and across situations (Corr & Matthews, 2020). Empirical research in Western countries (e.g., North America and Europe) commonly converges on five broad factors, with narrower traits within these factors: extraversion (sociable, chatty, active, forceful), agreeableness (cooperative, polite, sympathetic, trusting), conscientiousness (disciplined, industrious, organized, reliable), neuroticism (vs. emotional stability; anxious, easily upset, disposed to negative emotions), and openness or openness/intellect (inventive, curious, abstract thinking) (DeYoung et al., 2007; John & Srivastava, 1999). Each of the five factors can also be divided into two subfactors or *aspects*: extraversion (assertiveness, enthusiasm), agreeableness (compassion, politeness), conscientiousness (industriousness, orderliness), neuroticism (volatility, withdrawal), and openness/intellect (openness, intellect) (DeYoung et al., 2007). Although there is debate on the meaning of the Big Five as latent variables, and whether the scores on specific items within each factor stem from a common cause (Borsboom, 2023; Revelle, 2024), they form a practically useful framework.

#### 1.2.2. Agreeableness

The trait of agreeableness has had the strongest, most consistent associations with adoption of protective behaviors related to SARS-CoV-2 (Abdelrahman, 2022; Milad & Bogg, 2021; Nofal et al., 2020; Siritzky et al., 2022; Wright et al., 2022). Agreeableness is likely to be associated with protective behaviors because of an inclination to feel empathy for others, and so protect the health of others (the compassion aspect), and to be cooperative (the politeness aspect). This evidence directly justifies the first set of hypotheses:

**H1**. SARS-CoV-2 infection will be negatively associated with agreeableness.

**H2**. SARS-CoV-2 infection will be negatively associated with the compassion aspect of agreeableness.

**H3**. SARS-CoV-2 infection will be negatively associated with the politeness aspect of agreeableness.

#### 1.2.3. Conscientiousness

Conscientiousness also consistently has positive associations with protective behaviors (Abdelrahman, 2022; Bogg & Milad, 2020; Nofal et al., 2020; Wright et al., 2022). Conscientiousness is likely to be associated with adoption of preventive behaviors because those higher in conscientiousness tend to follow rules, and tend also to be responsible, disciplined, thorough, and careful, so may stick to rules and guidelines more consistently over time. Conscientiousness is also consistently associated with health enhancing behaviors (Bogg & Roberts, 2013; Jokela et al., 2013). This evidence informs hypothesis 4:

**H4**. SARS-CoV-2 infection will be negatively associated with conscientiousness.

#### 1.2.4. Openness

Higher openness (also openness/intellect) has been found to be positively associated with compliance with protective measures in several studies (Bogg & Milad, 2020 [indirectly]; Nofal et al., 2020; Qian & Yahara, 2020). However, in one study lower openness was correlated with higher general compliance (Wright et al., 2022). Openness may be related to greater knowledge about how a virus is transmitted and effective behaviors to protect oneself from infection, which has been associated with greater adoption of precautionary behavior in some studies but not others (Bish & Michie, 2010). Openness to trying new things may also make people more willing to adopt protective measures. There may also be an association between education level (which is related to openness) and adoption of behaviors, although this link is inconsistent across studies (Bish & Michie, 2010). Because of the inconsistent evidence of associations, particularly at the time of hypothesis preregistration, we make no formal hypotheses regarding openness.

#### 1.2.5. Extraversion

The association between extraversion and protective behaviors is less clear, with both positive (Qian & Yahara, 2020) and negative (Chan et al., 2021; Wright et al., 2022) associations with compliance. Extraversion may be associated with less adoption of recommended behaviors where this requires avoidance of other people, given the strong desire to socialize for those with high levels of extraversion.

Extraversion is one personality factor that has some evidence of an association with risk of infection. Christakis and Fowler (2010) used serological tests during the H1N1 2009 influenza pandemic, finding people with more friends and more centrally located in social networks (likely related to extraversion), on average contracted the flu earlier than others. Furthermore, Rolón et al. (2021) found the sociability facet of extraversion had a slight association with having contracted SARS-CoV-2 in the past (self-reported viral or antibody test). However, Brauer and Proyer (2022) note the small sample size of Rolón et al.’s (2021) study (*n* = 217), recommending replication with a higher-powered sample size, and the use of multimethod assessments. These previous results suggest more extraverted individuals will put themselves at greater risk of infection through socializing, implicating the particular aspect of enthusiasm, which focuses on the sociability element of extraversion:

**H5**. SARS-CoV-2 infection will be positively associated with extraversion.

**H6**. SARS-CoV-2 infection will be positively associated with the enthusiasm aspect of extraversion.

#### 1.2.6. Neuroticism

Neuroticism has the greatest range of associations with protective behaviors. Higher neuroticism has been positively associated with compliance with protective measures (e.g., Qian & Yahara, 2020), as has higher anxiety (Bults et al., 2011). However, lower neuroticism has been correlated with greater compliance in other studies (e.g., Siritzky et al., 2022; Wright et al., 2022). Those higher on worry and anxiety facets of neuroticism may be more inclined to adopt avoidance behaviors (Spielberger, 1979; Taylor, 2019), while those higher in volatility, characterized by anger and upset (DeYoung et al., 2007), may react more strongly against recommendations and rules. Given the range of evidence, we make no formal hypotheses regarding neuroticism.

#### 1.2.7. Optimism

Optimism is expressed both as a dispositional trait—an inclination to expect positive outcomes (Scheier & Carver, 1985)—and as situational state, which is context-dependent (Carver & Scheier, 1994). Defensive optimism or unrealistic optimism is an inclination to view situations as more positive than they realistically are, downplaying the negatives or significance of a situation (Gordeeva et al., 2020).

Constructive optimism (an element of dispositional optimism which emphasizes the role of effort and control in positively influencing events), has been associated with compliance with stay-at-home orders (Gordeeva et al., 2020), whereas defensive optimism and unrealistic optimism regarding negative life events have been associated with lower adoption of protective behaviors (Gordeeva et al., 2020; Oljača et al., 2020; Salgado & Berntsen, 2021), with evidence that this is mediated by underestimation of personal COVID-19 risk (Fragkaki et al., 2021). The evidence now suggests a negative relationship between dispositional optimism and infection, but a positive relationship between defensive or unrealistic optimism and infection. However, given the limited information on associations between optimism and COVID-19 protective behaviors at the time of hypothesis preregistration, we make no formal hypothesis, and analyses of an association between SARS-CoV-2 infection and optimism are exploratory only.

#### 1.2.8. Risk taking and risk perception

People vary in their general tendency to perceive life activities as risky, and their willingness to take risks. Risk attitudes also differ within people across domains, for example, between health risks and financial risks (Blais & Weber, 2006). Further, individuals vary in their risk perception regarding specific pandemics, such as COVID-19. Some see virtually no risk (Rosenstock, 1960), while others perceive a threat well above the actual threat level (Taylor, 2019). Pandemic-specific risk perceptions, perceived effectiveness of protective behaviors, and knowledge about virus transmission are important determinants of tendency to take protective action (Bish & Michie, 2010; Rosenstock, 1960; Soper, 1919).

Risk perception may also be predicted by personality traits and demographic variables. Those higher in neuroticism, emotionality, and anxiety in particular, have greater perception of COVID-19 risk and fear (Garbe et al., 2020; Lippold et al., 2020; Tagini et al., 2021), while higher openness predicts perception of less risk (Tagini et al., 2021). Men have been found to have lower levels of risk perception than women generally (Gustafson, 1998), and specifically in relation to COVID-19 (Lippold et al., 2020; Tagini et al., 2021). Men also have higher levels of risk taking than women on average (Byrnes et al., 1999; Zuckerman & Kuhlman, 2000).

Inclination to take risks has been negatively associated with compliance with protective measures (Miguel et al., 2021; Wright et al., 2022). Fear and perception of risk, in relation to COVID-19, has been positively associated with compliance (Abdelrahman, 2022; Horner et al., 2023). We form two hypotheses, given the clear pre-COVID and COVID evidence on the effects of risk perception and risk taking on health protective behaviors:

**H7**. SARS-CoV-2 infection will be negatively associated with risk perception.

**H8**. SARS-CoV-2 infection will be positively associated with risk taking.

Importantly, some of the bivariate correlations between personality and protective behaviors are modest, around 0.10 (e.g., Nofal et al., 2020), and some disappear once control demographics, political beliefs, or country-level political and cultural variables, are taken into account (e.g., Milad & Bogg, 2021; Siritzky et al., 2022).

### 1.3. The current study

Early work documented associations between personality and COVID-19 related protective behaviors, but it remains unknown which personality characteristics may impact the risk of *actually* contracting SARS-CoV-2. Further, studies reporting the effect of personality on contracting SARS-CoV-2 have had modest sample sizes, or used self-reported infection, potentially distorting results. For example, those higher on neuroticism are more likely to self-report virus symptoms (Milad & Bogg, 2021). Further, perceived symptoms are only moderately predictive of actual laboratory confirmed COVID infection (Matta et al., 2022; Popovych et al., 2021), and antigen tests have in the past varied greatly in their accuracy, particularly for asymptomatic infection (Dinnes et al., 2022). More generally, some argue that psychology should include more research that includes objective outcomes such as physiological measurement (Baumeister et al., 2007), and that there is an overreliance on self-reports, which can create common method bias, inflating correlations between self-report measures (Podsakoff et al., 2003). Further, there is considerable variation in rates of infection by geographical area and demographic subgroup, so we included key demographics: region of the United States, age group, sex, race, ethnicity, education, and urban versus rural area of residence.

The primary aim of this research was to determine the influence of personality on the likelihood of contracting SARS-CoV-2, using rigorous methods that address the limitations of self-report common method bias, self-report of infection, small sample sizes, or samples where asymptomatic infection may be mistaken for lack of infection. Given associations between personality and adoption of protective behaviors, we formulated several hypotheses.

### 1.4. Hypotheses

The hypotheses were that people would be more likely to test positive for SARS-CoV-2 antibodies if they were: (1) lower on agreeableness, (2) lower on the compassion aspect of agreeableness, (3) lower on the politeness aspect of agreeableness, (4) lower on conscientiousness, (5) higher on extraversion, (6) higher on the enthusiasm aspect of extraversion, (7) lower on risk perception, and (8) higher on risk taking. Hypotheses were all preregistered: https://osf.io/6yxje/?view_only=ec2c9e3af4f84c3bb32031a76bb3d675 and https://osf.io/tsgpy/?view_only=e68b03aad4d04d85b541dc0958cc4cbb. The first preregistration outlined our study hypotheses and general analytic plan before personality data collection began. The second preregistration was created after personality data collection had commenced to specify additional modeling details (e.g., inclusion of multilevel logistic models) based on the structure of the serological testing data. A summary table comparing preregistered and conducted analyses, including deviations and exploratory additions, is provided in the Supplement (Table S11).

## 2. Method

### 2.1. Participants and procedure

Participants (*n* = 6158, 53 % female, age *M* = 50.63, *SD* = 15.33) were adults originally accepting an invitation to participate in research on seroprevalence of SARS-CoV-2 in the United States (*n* = 9029), and reflected characteristics of the U.S. general adult population. These participants had not previously been diagnosed with COVID-19, however, 4.6 % were found to have been infected with SARS-CoV-2 (Kalish et al., 2021). After first blood sample collection, these participants were invited to participate in the current research by completing personality questionnaires online. Sample demographics (*n* = 6158) are compared with 2020 U.S. census demographics and Behavioral Risk Factor Surveillance System demographics (BRFSS; United States Census Bureau, 2020) in Table 1. Participant race (e.g., Black only, White only), ethnicity, and sex categories are outlined in Kalish et al. (2021), and more details on the broader sampling process are provided in Fig. 1 from Kalish et al. (2021).

**Fig. 1.**
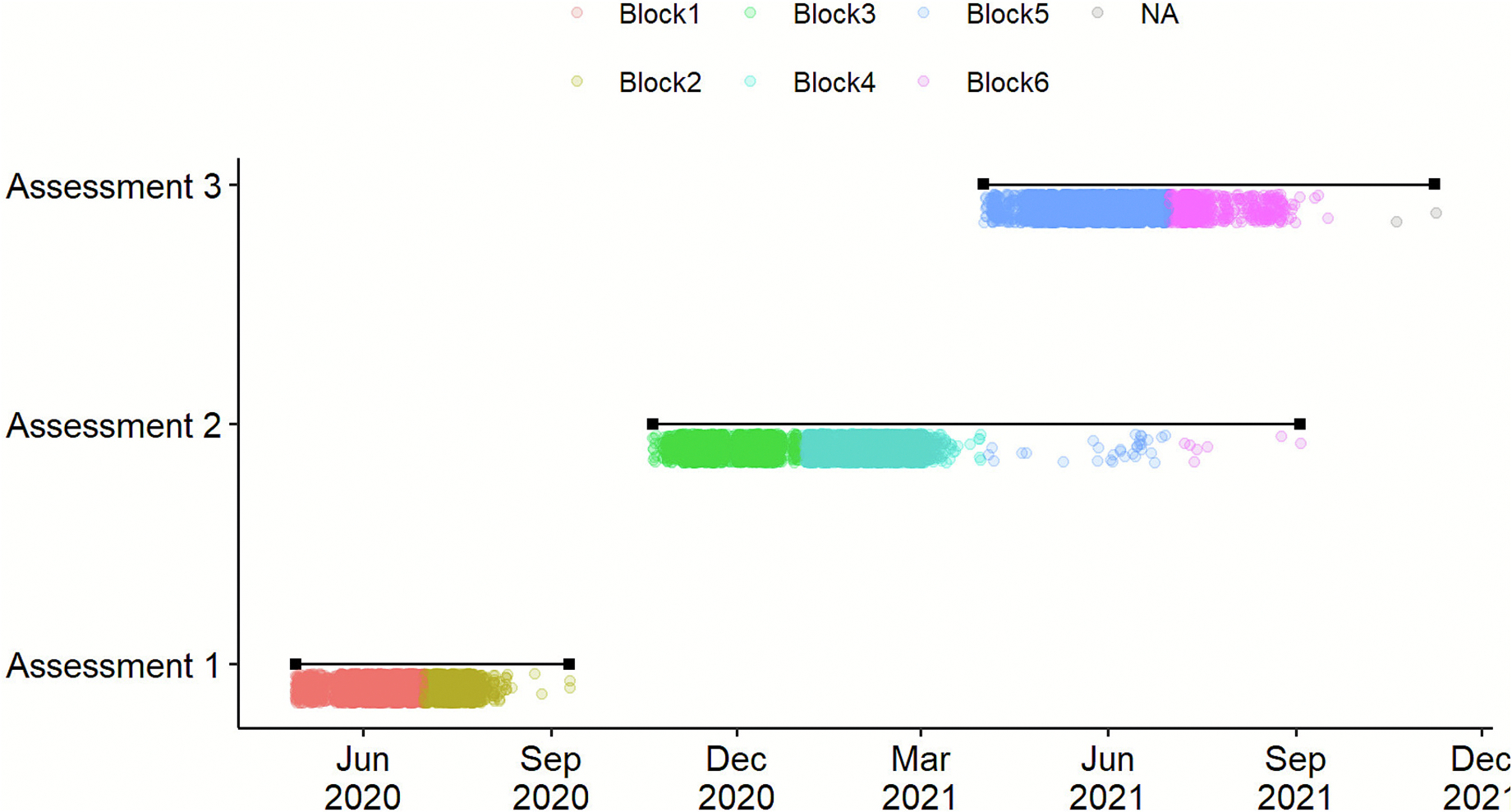
Dates of seropositivity assessment by assessment period.

Blood samples for serological tests were collected up to three times (4038 had 3 tests, 1314 had 2 tests, 806 had 1 test), with assessments approximately six months apart for each person, although as a group there was some overlap between the assessment time periods. Table 2 and Fig. 1 depict the distribution of blood test dates, with most samples collected for assessment period 1 from May to July 2020, for assessment period 2 from October 2020 to March 2021, and for assessment period 3 from April 2021 to August 2021.

Participants gave informed consent to have their results used for research. They were not paid, but were offered a personalised report interpreting their personality results, after all blood samples had been provided and questionnaires completed. This research was approved by the NIH Institutional Review Board and conducted in accordance with the provisions of the Declaration of Helsinki and Good Clinical Practice guidelines. The serosurvey clinical trial element of the research has the reference number: ClinicalTrials.gov NCT04334954.

### 2.2. Blood tests and assessment of Seropositivity

The majority of participants gave 80 μl blood samples using a Mitra microsampling kit, drawing blood from the fingertip, and sent the sample by mail. A small number of participants also provided 18 ml of blood through venipuncture. Blood samples underwent serological assay for antibodies using enzyme-linked immunoassay (ELISA). To determine the threshold measurements for seropositivity, 56 true positive and 300 true negative blood samples were assayed, and seropositivity was defined as mean optical density plus three standard deviations as previously described (Kalish et al., 2021; Klumpp-Thomas et al., 2021). Categorization of seropositivity required both spike protein positivity and receptor binding domain (RBD) positivity, for either IgG or IgM, with IgA providing additional discrimination, but without being sufficient on its own to categorize a sample as seropositive. Specificity was 100 % (95 % CI [96.4 %, 100 %]) and sensitivity was 100 % (95 % CI [76.8 %, 100 %]) (Klumpp-Thomas et al., 2021). Importantly, serological testing can distinguish between a COVID-19 vaccine immune system response and SARS-CoV-2 immune system response, as SARS-CoV-2 infection produces antibodies to the nucleocapsid protein, whereas the vaccine does not. The primary outcome was seropositivity, indicating SARS-CoV-2 infection. Antibody testing was the most appropriate approach, given our interest in who had been infected over the preceding six months, not just the narrow window of current infection (viral testing). For further detail on blood sampling and serological assay procedures, see Kalish et al. (2021) and Klumpp-Thomas et al. (2021).

### 2.3. Personality measures

We used three personality measures: the Big Five Aspect Scales (BFAS; DeYoung et al., 2007); the Domain-Specific Risk-Taking scale (DOSPERT; Blais & Weber, 2006), measuring risk perception and risk taking; and the Life Orientation Test Revised (LOT-R; Scheier et al., 1994), measuring optimism. Completion of each measure was voluntary, so completion numbers ranged from *n* = 4721 for the DOSPERT, to *n* = 4739 for the BFAS. Also included were key demographic factors: region of the United States, age group, sex, urban/rural, race, ethnicity, and education.

#### 2.3.1. The Big Five Aspect Scales

The Big Five Aspect Scales (BFAS; DeYoung et al., 2007) is a 100-item self-report measure of the Big Five factors: neuroticism, extraversion, openness/intellect, agreeableness, and conscientiousness. Included beneath each factor are two *aspect* subscales: volatility, withdrawal, enthusiasm, assertiveness, intellect, openness, compassion, politeness, industriousness, and orderliness. Items were answered on a 5-point Likert-type scale, from 1 = *Strongly Disagree* to 5 = *Strongly Agree*.

#### 2.3.2. Domain-Specific Risk-Taking scale

The Domain-Specific Risk-Taking scale (DOSPERT; Blais & Weber, 2006) is a 30-item measure of risk taking and risk perception, each having subscales for five different domains of life: ethical (e.g., passing off somebody else’s work as your own), financial (e.g., investing 5 % of your annual income in a very speculative stock), health/safety (e.g., driving a car without wearing a seatbelt), recreational (e.g., going down a ski run that is beyond your ability), and social (e.g., speaking your mind about an unpopular issue in a meeting at work). Respondents answer the questions twice, once responding to “Please indicate how risky you perceive each situation”, on a 7-point Likert-type scale, from 1 = *Not at all Risky* to 7 = *Extremely Risky*, and once responding to “Please indicate the likelihood that you would engage in the described activity or behavior if you were to find yourself in that situation”, on a 7-point Likert-type scale, from 1 = *Extremely Unlikely* to 7 = *Extremely Likely*.

#### 2.3.3. The Life Orientation Test Revised

The Life Orientation Test Revised (LOT-R; Scheier et al., 1994) is a 10-item measure of dispositional optimism, with three positively scored items, three reverse scored items, and four filler items. Items are answered on a 5-point Likert-type scale, from 1 = *Strongly Disagree* to 5 = *Strongly Agree*.

Participants were allowed to skip questions; as a result, some participants are missing information for some scales. For each model estimated (described below), only participants who had completed the relevant personality measure were included. No imputation was performed for personality measures.

### 2.4. Data analytic approach and preregistered analyses

We proposed a series of analyses using data previously analyzed by Kalish et al. (2021) and data that was yet to be collected. Logistic multilevel modeling was designated as the primary inferential approach for testing associations between personality and infection status, with bivariate correlations provided as descriptive statistics.

#### 2.4.1. Personality trait relationship with SARS-CoV-2 infections

We assessed the zero-order relationships between traits and seropositivity, using a standard Pearson product-moment correlation (equivalent to the point-biserial in this case). Seropositivity was a dichotomous variable indicating whether the participant ever tested seropositive to SARS-CoV-2 during the study (1) or never (0). Participants were weighted in accordance with U.S. Census demographic categories, to best approximate representative effect sizes. We report Pearson’s *r* for bivariate associations and odds ratios (ORs) from logistic regression models. These metrics align with our preregistration, are standard in personality–epidemiology research, and facilitate comparison with prior work. While other effect size metrics (e.g., Cohen’s *d*) are mathematically related to *r* and OR, we chose to retain these formats to maintain interpretability and consistency. We also report both uncorrected *p-*values and Holm corrected *p*-values (Holm, 1979) for transparency.

#### 2.4.2. Personality prediction of SARS-CoV-2 infection overall and beyond demographics

We fit binomial logistic multilevel models (MLM) with assessments (Level 1) nested within person (Level 2). The outcome was whether the participant tested seropositive from infection (i.e., not vaccination), measured separately at each assessment. Level 1 predictors were the estimated prevalence of SARS-CoV-2 in participants’ county of residence on the date of assessment. We used county-level SARS-CoV-2 prevalence as a covariate rather than state-level prevalence because county estimates provide finer-grained measures of local infection risk. This approach better captures the variation in potential exposure environments experienced by participants and reduces the ecological imprecision inherent in state-level aggregates.

Level 2 predictors were person-level personality traits and demographics (age, sex, race, ethnicity, urban/rural residence, and education). While many covariates are objectively not caused by personality (e.g., age), education has a complex bi-directional causal relationship with traits, notably openness, that unfolds over the lifespan (Furnham & Cheng, 2016). We chose to include education here as a covariate because it is plausibly a common cause of both mid- and late-life openness and infection risk, in part through its association with health literacy and related behaviors. More specifically, given that personality was measured in adulthood after most participants’ formal education was completed, education is unlikely to operate as a mediator or collider in this context.

One model was fit for each trait and intercepts were allowed to vary across participants. We provide odds ratios to aid interpretation. We did not include all Big Five traits in a single model because of substantial intercorrelations among traits, which can lead to multicollinearity and unstable parameter estimates. Modeling each trait separately provides more stable and interpretable estimates of its overall association with infection risk.

We examined the intraclass correlation coefficient (ICC) for seropositivity at the U.S. state level. The observed ICC was approximately 0.00, indicating that none of the variance in seropositivity was attributable to differences between states. This value is consistent with prior work showing minimal personality-related variance between states (Elleman et al., 2018) and supports the decision not to model state-level clustering in our primary analyses.

#### 2.4.3. Personality trait prediction of change in risk over time

Longer evasion of infection may be influenced by personality or interaction with the changing nature of the pandemic. Including time as a categorical variable accounts for variation in infection risk across distinct data collection periods, defined by substantial changes in local and national pandemic conditions (e.g., new variants, public health policy shifts, changes in vaccination coverage). We tested whether personality traits predicted change in risk over time, modifying the multilevel models (Section 2.4.2) by including time as a predictor at Level 1, with time operationalized as a categorical variable in discrete three-month blocks (see Supplement Section 1.6 for detail). This allowed control for period-specific baseline differences in exposure likelihood and testing of whether personality–infection associations were consistent across these epidemiological contexts, rather than confounded by temporal shifts in infection dynamics.

#### 2.4.4. Preliminary analysis and descriptive statistics

Descriptive statistics for all personality variables are shown in Table 3 (Table S1 of the Supplement for further detail). Means for the openness/intellect factor (3.94) and the intellect aspect (4.03) were higher than the two BFAS validation (DeYoung et al., 2007) norm group means for the openness/intellect factor (3.72, 3.47) and intellect aspect (3.70, 3.39). The openness/intellect standard deviation was also smaller than the other scales.

### 2.5. Transparency and openness

All data and analysis codes are provided on the Open Science Framework site: https://osf.io/z82pv/?view_only=296e5c3e9cda4344b393352eec566ead Analysis plans were preregistered: https://osf.io/6yxje/?view_only=ec2c9e3af4f84c3bb32031a76bb3d675. We describe determination of our sample size, all manipulations, and all measures in the study. There were no data exclusions. Full personality questionnaire versions used for research materials are in the source articles cited. Data were analyzed using the psych package (Revelle, 2023) in R (R Core Team, 2023).

## 3. Results

### 3.1. Seropositivity per time period

There were 513 participants out of 5987 (8.6 %) who were seropositive at assessment time 1, 2413 out of 5162 (47 %) at assessment time 2, and 2423 out of 4399 (55 %) at assessment time 3 (Table 2). Seropositivity rates for each of the six blocks are shown in Table S6 of the Supplement. Seropositivity totals for each time period are not strictly cumulative, because a small proportion of participants (<2 %) tested seropositive at one time, but seronegative later, as antibodies do not always remain indefinitely (Table 4). Someone can only be infected for the first time once, so rates of first infection for each time period (for males and females) are shown in Table S2 of the Supplement. First time infection rates were 8.6 % (513 of 5987) for time point 1, 40.2 % (2077 of 5162) for time point 2, and 9.9 % (436 of 4399) for time point 3. Females had higher rates of infection overall (53 % vs 45 %) and for time periods 1 and 2.

Correlations between personality traits and covariates are shown in the Supplement, Table S3. Consistent with previous research, males were lower on agreeableness (*r* = −0.25, *p* < .001), higher on risk taking (*r* = 0.21, *p* < .001), and lower on risk perception (*r* = −0.27, *p* < .001).

Correlations between seropositivity, prevalence, and covariates are shown in Table 5. Noteworthy results are the positive correlation between seropositivity and county prevalence (*r* = 0.05, *p* < .001), and negative correlation between seropositivity and being male (*r* = −0.08, *p* < .001). The sex difference in seropositivity is likely to have caused the correlation, before demographic controls, of seropositivity with agreeableness (Table 6).

### 3.2. Personality prediction of SARS-CoV-2 infection beyond demographics

After controlling for demographic factors, we found that no personality traits emerged as predictive of SARS-CoV-2 infection after Holm correction (a strict threshold). The openness/intellect factor and narrower intellect aspect were significant before correction (Table 6; Table S4 of Supplement for more detailed results), but these were not pre-registered hypotheses.

We conducted additional analyses, departing from preregistration, analyzing the relationship of personality traits to SARS-CoV-2 seropositivity beyond demographic controls, but omitting county COVID-19 prevalence. This is justified by the many asymptomatic and mild cases that never get detected (Kalish et al., 2021), with underestimation likely varying by state and county, plus lack of knowledge about precise infection date. These issues were not foreseen when registering the analyses. The new analyses showed that the openness/intellect factor had a significant but small negative association with seropositivity, after Holm correction (standardized OR = 0.85, *p* = .050; see Table 6, and Table S5 of the Supplement), comparable to a Pearson’s correlation of −0.05.

### 3.3. Personality trait relationship with SARS-CoV-2 infections

Analysis of correlations between traits and seropositivity without demographic controls—testing seropositive from infection (not vaccination) at any point during the study (1) or never (0)—found 13 significant but very small correlations before Holm correction (Table 6). However, there were only three significant results after Holm correction, with agreeableness having a *positive* correlation with seropositivity, and both the broad openness/intellect factor and narrower openness aspect having a negative correlation with seropositivity.

### 3.4. Other analyses

#### 3.4.1. Personality trait prediction of change in risk over time

We analyzed association of personality with change in risk over time. None of the personality traits were associated; no traits moderated change in seropositivity risk over time. Further detail on these analyses and results is in the Supplement (Tables S6 and S7).

#### 3.4.2. Mediation analysis

Vaccination status was included as a mediator in our preregistered models, given its potential to influence infection risk. Full results are presented in Table S8. Associations between personality and vaccination were small, and inclusion of vaccination as a mediator did not substantively alter the pattern of personality–infection associations.

#### 3.4.3. Exploratory survival models

Baseline and Cox proportional hazards models were used to assess the relationship of the continuous personality variables to survival odds (Supplement Tables S9 and S10). These Cox proportional hazards models serve as sensitivity analyses, complementing our primary logistic models. Across both analytic approaches, the pattern of results and substantive conclusions were consistent: after controlling for demographics, higher scores on the openness/intellect factor (*p* = .001), and openness aspect (*p* < .001) were found to be associated with less likelihood of early infection. These analyses were not preregistered.

## 4. Discussion

This study aimed to determine the relationship between personality traits and SARS-CoV-2 seropositivity. A small but significant negative association was found between the openness/intellect factor and seropositivity when county prevalence of SARS-CoV-2 was omitted, and also before the strict corrections for multiple comparisons (not preregistered). The openness/intellect factor and openness aspect had significant associations with survival odds, after controlling for demographics. Contrary to predictions, after control variables and corrections for multiple tests, no other substantial relationships were found between traits and seropositivity.

To contextualize the magnitude of effects, the largest statistically significant associations (e.g., openness/intellect r ≈ −0.05) correspond to changes in infection probability of <1 percentage point across the observed range of scores. This reinforces that, while statistically detectable in a large sample, these effects are very small in practical terms. There are several potential explanations for the general lack, or small size, of associations. First, many of the associations previously found between protective behaviors and personality have been quite small. The relationship between protective behaviors and contracting the virus is also imperfect, further diluting the indirect pathway from traits to infection. Second, some pandemic situations may have had far greater impact on the probability of infection than the behaviors people chose within those situations. For example, if one is a bus driver, whether or not one wears a mask may not strongly influence whether one ultimately contracts COVID-19. Third, some situations in the pandemic are strong situations, constraining the degree to which people’s personalities are expressed through behavior (Cooper & Withey, 2009). Examples in the pandemic would be state-enforced shelter-in-place mandates, or workplaces requiring staff contact with others (e.g., hospitals). Fourth, protective behaviors have varying effectiveness, so personality may predict adoption of behaviors but not evasion of infection. Complete avoidance of other people would clearly avoid infection. In contrast, disinfecting surfaces, later recognized as lower risk for infection, may have had limited benefits (Lewis, 2021). In the United States, face-mask wearing was initially not recommended for the general public, but advice evolved to cloth masks, surgical masks, then to the most effective medical grade fitted masks (Boulos et al., 2023).

One of the strongest predictors of seropositivity is known exposure to COVID-19 (Kalish et al., 2021). We must take into account level of exposure based on location—SARS-CoV-2 prevalence in the broader geographic location, but also specific location risks from crowd density, at school, work, or when commuting. A person whose work requires close contact with colleagues and customers, catches a train to work, and lives with six people will have higher risk, regardless of their choices.

Our research found a clear sex difference in SARS-CoV-2 infection rates, with females more likely to test positive than males (53 % vs 45 %). This contrasts with large-scale worldwide research, showing slightly higher seroprevalence in males (Rostami et al., 2021), but was consistent with United States research (Danielsen et al., 2022). This is surprising given females have higher levels than males of general compliance with protective measures in relation to COVID-19 (Gordeeva et al., 2020; Wright et al., 2022), and regarding previous pandemics (Bish & Michie, 2010; Moran & Del Valle, 2016). It is possible that differences between typical male and female societal and work roles influenced exposure to SARS-CoV-2.

There were small negative associations between the openness/intellect factor and seropositivity, that remained after controlling for level of education, and do not appear to be solely due to the narrower intellect aspect. The small negative association might be related to greater knowledge about how a virus is transmitted and effective behaviors to protect oneself, which has been associated with greater adoption of precautionary behavior in some studies but not others (Bish & Michie, 2010). Openness to trying new things may also make people amenable to protective measures.

These associations may also be due to a cultural-political factor, as lower openness is associated with more right-wing or conservative political views (McCrae, 1996), which in turn may have been associated with lower adoption of protective measures in the United States through political party affiliation and associated beliefs (Howard, 2022). It is difficult to generalize to other countries, but results indicate the potential importance of *beliefs* in predicting infection.

However, inconsistent with some studies finding associations between personality and protective behaviors, we did not even find clear personality-infection correlations.

### 4.1. Limitations

First, as Kalish et al. (2021) point out, although their sample is matched to U.S. general population demographics, it is based on volunteers, which may create selection bias. Our sample was a subset of that larger sample, and had higher average education levels than the U.S. general population. This may have reduced variance of both the openness/intellect scale and of preventive behaviors. However, the sample was representative for age, sex, race, and ethnicity.

Second, self-reported protective behaviors such as mask-wearing were not measured. Although self-report can be biased, this would have allowed a direct test of the proposed causal mechanism to infection. However, given the lack of associations between personality and infection, this is somewhat of a moot point.

Third, although we measured Big Five personality, with narrower aspects, risk taking and risk perception, and optimism, we did not measure other individual differences such as Dark Triad traits (e.g., psychopathy, grandiose narcissism) which have been associated with reduced compliance to COVID-prevention behavior (Blanchard et al., 2023; Pourdehghan et al., 2025).

## 5. Conclusion

In contrast to expectations, openness/intellect was associated with SARS-CoV-2 infection, but other personality traits had no measurable association. Situational and demographic variables were associated with SARS-CoV-2 infection. These findings contrast with the logically plausible view that those with the ‘right’ personality will have lower chance of infection via more frequent adoption of protective behaviors. Future research should focus on understanding the reasons for different infection rates in different demographics, particularly male versus female.

## Supplementary Material

1

## Figures and Tables

**Table 1:** Sample demographic, U.S. population census, and BRFSS characteristics.

Characteristic	*n* (%)	BRFSS (%)	U.S. census 2020 (%)

Age^a^	50.63 (15.33) [18–92]		38.8^b^ (median)
Sex (female)	3250 (52.8 %)	54.76 %	50.92 %
Race			
American Indian/Alaska Native	154 (2.5 %)		1.12 %
Asian only	354 (5.7 %)		6.00 %
Black only	510 (8.3 %)	8.87 %	12.40 %
Multiple races	134 (2.2 %)		10.21 %
Other	112 (1.8 %)		8.42 %
Pacific Islander only	8 (0.1 %)		0.21 %
White only	4886 (79 %)	81 %	61.63 %
Ethnicity			
Hispanic	978 (16 %)	8.53 %	18.73 %^c^
Non-Hispanic	5180 (84 %)	91.47 %	81.27 %^c^
Education			
≤High school	169 (2.7 %)	34.79 %	
College	856 (14 %)	27.53 %	
≥College	5133 (83 %)	37.68 %	37.5 %^d^
Residence			
Rural	335 (5.4 %)	15.1 %	20.0 %
Urban	5823 (95 %)	84.9 %	80.0 %

Note. *N* = 6158, including 1419 participants who did not complete any personality questionnaires. BRFSS = Behavioral Risk Factor Surveillance System survey and is a large health-focused telephone survey of U.S. residents.

a Age mean (SD) [Minimum-Maximum].

b Includes under 18.

c Hispanic or Latino are grouped together in U.S. Census.

d https://www.statista.com/statistics/184260/educational-attainment-in-the-us/.

**Table 2:** Seropositivity assessment time periods.

	Time period 1	time Period 2	Time period 3

Date range	29 April 2020–10 Sep 2020	21 Oct 2020–3 Sep 2021	1 April 2021–8 Nov 2021
Known	5987	5162	4399
Unknown (missing)	171	996	1759
Seropositive	513 (8.6 %)	2413 (46.7 %)	2423 (55.1 %)
Seronegative	5474	2749	1976

Note. *N* = 6158, including 1419 participants who did not complete any personality questionnaires. For this table, seropositive totals for each time period are those who were seropositive when tested in that time period—these figures are mostly but not strictly cumulative, as a small proportion of participants may be seropositive, but seronegative later. Other descriptive and analysis results will describe participants in terms of whether they were *ever* seropositive. Percentages here are calculated from total seropositives out of the available sample for that time period (excludes participants not sero-tested for that period).

**Table 3:** Descriptive statistics for scales measuring traits (factors, aspects, and specific domains).

Personality trait	Mean	*SD*	Cronbach’s alpha

Extraversion	3.61	0.47	0.88
Assertiveness	3.58	0.56	0.87
Enthusiasm	3.64	0.57	0.85
Agreeableness	4.04	0.36	0.84
Compassion	4.11	0.43	0.86
Politeness	3.98	0.46	0.76
Conscientiousness	3.66	0.46	0.86
Industriousness	3.63	0.54	0.85
Orderliness	3.68	0.55	0.81
Neuroticism	2.41	0.56	0.92
Volatility	2.39	0.62	0.90
Withdrawal	2.42	0.61	0.87
Openness/Intellect	3.94	0.37	0.81
Intellect	4.03	0.46	0.82
Openness	3.86	0.48	0.76
General risk perception	4.70	0.58	0.86
Ethical	5.61	0.83	0.66
Financial	5.05	0.81	0.75
Health	5.52	0.86	0.71
Recreational	4.70	1.02	0.79
Social	2.60	0.75	0.68
General risk taking	2.78	0.60	0.82
Ethical	1.61	0.67	0.54
Financial	2.31	0.80	0.67
Health	2.10	0.93	0.62
Recreational	2.71	1.25	0.79
Social	5.15	0.92	0.68
Optimism	3.75	0.50	0.79

Note. Big Five personality (*n* = 4739), risk perception (*n* = 4731), risk taking (*n* = 4721), optimism (*n* = 4738).

**Table 4:** Totals of combinations of seropositive, seronegative, and missing data.

Assessment number			
	
1st (baseline)	2nd (6 month)	3rd (12 month)	*N*

1	1	1	268
1	1	0	1
1	1	NA	67
1	0	1	67
1	0	0	2
1	0	NA	12
1	NA	1	22
1	NA	NA	74
0	1	1	1567
0	1	0	19
0	1	NA	402
0	0	1	370
0	0	0	1744
0	0	NA	485
0	NA	1	52
0	NA	0	174
0	NA	NA	661
NA	1	1	63
NA	1	NA	26
NA	0	1	7
NA	0	0	30
NA	0	NA	32
NA	NA	1	7
NA	NA	0	6
Total tested seropositive for first time at each assessment time point			Total ever tested seropositive
513	2077	436	3026
Total never tested seropositive			
			3132

Note. *N* = 6158. 1 = seropositive, 0 = seronegative, NA = participant not tested. Participants with 3 sero-tests (*n* = 4038); participants with 2 sero-tests (*n* = 1314); participants with 1 sero-test (*n* = 806).

**Table 5:** Correlations between seropositivity, prevalence, and covariates.

	1	2	3	4	5	6	7	8	9	10	11	12

1. COVID positive												
2. County prevalence	**0.054**											
3. Age	**0.034**	**−0.058**										
4. Sex (male)	**−0.080**	0.009	**−0.027**									
5. Urban vs rural	−0.001	**−0.046**	**0.056**	0.011								
6. American Indian/Alaska Native vs White only	0.021	0.006	**−0.038**	−0.020	0.012							
7. Asian only vs White only	−0.006	**−0.049**	**−0.096**	**−0.030**	**−0.059**	**−0.040**						
8. Black only vs White only	**0.026**	−0.005	**−0.089**	**−0.104**	**−0.067**	**−0.048**	**−0.074**					
9. Multiple races vs White only	−0.020	−0.023	**−0.068**	−0.005	−0.016	−0.024	**−0.037**	**−0.045**				
10. Other vs White only	−0.003	0.004	**−0.081**	0.003	**−0.027**	−0.022	**−0.034**	**−0.041**	−0.020			
11. Ethnicity Hispanic	0.004	**0.065**	**−0.197**	0.003	**−0.085**	**0.098**	**−0.094**	**−0.069**	**0.045**	**0.227**		
12. Education (<high school)	−0.006	0.003	−0.009	0.018	**0.043**	0.005	−0.020	**−0.025**	−0.011	0.014	**0.025**	
13. Education (college)	−0.005	0.019	**0.026**	0.013	**0.048**	**0.026**	**−0.065**	−0.005	0.014	0.009	**0.028**	**−0.067**

Note. *N* = 6158, including 1419 participants who did not complete any personality questionnaires. Seropositivity values taken from participants’ last day of data collection, only for purpose of this correlation table [as COVID prevalence at later time points was more varied and more reflective of long-term trends]. Categorical variables are dummy coded, with most populated group serving as the reference group (Women, Urban dwellers, White only, Non-Hispanic, and College degree or more educated). Sex was coded male = 1, female = 0. All significant correlations are bold: *p* < .05 (*r* ≥ |0.025|), *p* < .01 (*r* ≥ |0.033|), *p* < .001 (*r* ≥ |0.042|).

**Table 6:** Correlations of personality traits (factors, aspects, domains) with SARS-CoV-2 seropositivity.

	Relationship with seropositivity beyond demographics (standardized effects)				Relationship with seropositivity beyond demographics (omitting county seroprevalence) (standardized effects)				Bivariate correlation with seropositivity		
			
			*p* -Value				*p*-Value			*p*-Value	
								
Personality trait factor, aspect, or domain	Est	OR	Raw	Correct	Est	OR	Raw	Correct	*r*	Raw	Correct

Extraversion	−0.19	0.83	0.730	>0.999	−0.04	0.97	0.504	>0.999	−0.04	0.006	0.147
Assertiveness	−0.12	0.88	0.818	>0.999	−0.05	0.95	0.333	>0.999	−0.04	0.004	0.100
Enthusiasm	−0.03	0.97	0.954	>0.999	−0.01	0.99	0.870	>0.999	−0.02	0.083	>0.999
Agreeableness	0.82	2.27	0.108	>0.999	0.10	1.10	0.082	>0.999	**0.04** *	0.001	0.033
Compassion	0.40	1.49	0.470	>0.999	0.10	1.10	0.079	>0.999	0.04	0.003	0.065
Politeness	0.83	2.29	0.100	>0.999	0.06	1.06	0.276	>0.999	0.03	0.028	0.486
Conscientiousness	0.32	1.38	0.521	>0.999	0.01	1.01	0.788	>0.999	0.00	0.851	>0.999
Industriousness	−0.28	0.76	0.603	>0.999	−0.06	0.95	0.299	>0.999	0.01	0.616	>0.999
Orderliness	0.87	2.40	0.080	>0.999	0.08	1.08	0.140	>0.999	−0.01	0.407	>0.999
Neuroticism	0.51	1.67	0.315	>0.999	0.06	1.06	0.256	>0.999	0.01	0.315	>0.999
Volatility	0.31	1.36	0.535	>0.999	0.04	1.04	0.433	>0.999	0.01	0.360	>0.999
Withdrawal	0.65	1.92	0.243	>0.999	0.07	1.07	0.203	>0.999	0.01	0.368	>0.999
Openness/Intellect	−1.41	0.24	0.008	0.235	**−0.17** *	0.85	0.002	0.050	**−0.05** **	<0.001	0.005
Intellect	−1.18	0.31	0.027	0.695	−0.13	0.88	0.019	0.489	−0.03	0.044	0.702
Openness	−1.03	0.36	0.052	>0.999	−0.14	0.87	0.009	0.234	**−0.05** **	<0.001	0.003
General risk perception	1.25	3.47	0.021	0.565	0.10	1.10	0.088	>0.999	0.03	0.007	0.152
Ethical	1.05	2.86	0.040	0.994	0.09	1.10	0.096	>0.999	0.01	0.285	>0.999
Financial	0.66	1.94	0.197	>0.999	0.05	1.05	0.370	>0.999	0.02	0.179	>0.999
Health	0.49	1.63	0.355	>0.999	0.06	1.06	0.324	>0.999	0.03	0.023	0.467
Recreational	0.53	1.70	0.343	>0.999	0.05	1.05	0.403	>0.999	0.03	0.016	0.327
Social	0.62	1.85	0.247	>0.999	0.07	1.07	0.182	>0.999	0.02	0.055	0.823
General risk taking	−0.59	0.55	0.287	>0.999	−0.03	0.97	0.620	>0.999	−0.02	0.174	>0.999
Ethical	0.28	1.32	0.578	>0.999	0.10	1.11	0.057	>0.999	0.02	0.207	>0.999
Financial	−0.83	0.43	0.102	>0.999	−0.07	0.93	0.179	>0.999	−0.01	0.298	>0.999
Health	0.00	1.00	0.996	>0.999	0.02	1.02	0.661	>0.999	−0.01	0.584	>0.999
Recreational	−0.30	0.74	0.601	>0.999	−0.01	0.99	0.852	>0.999	−0.02	0.209	>0.999
Social	−0.81	0.45	0.133	>0.999	−0.10	0.90	0.057	>0.999	−0.03	0.025	0.470
Optimism	−0.48	0.62	0.390	>0.999	−0.02	0.98	0.681	>0.999	−0.03	0.027	0.486

Note. Big Five personality (*n* = 4739), risk perception (*n* = 4731), risk taking (*n* = 4721), optimism (*n* = 4738). Correlations calculated using population weights to best approximate U.S. sex, age, education, and ethnicity. Correct = Corrected *p*-values, resulting from a Holm (1979) correction. Est = estimate. OR = Odds Ratio. All significant correlations after corrections are bold:

* *p* < .05,

** *p* < .01.

## Data Availability

All data and analysis codes are provided on the Open Science Framework site, with links provided in the Methods section. We also provide a PDF of analysis results and R code.

## Appendix A. Supplementary data

Supplementary data to this article can be found online at https://doi.org/10.1016/j.paid.2025.113549.
