# Supplementary material for: The effect of personality on likelihood of contracting SARS-CoV-2 in the United States
Source: Pers Individ Dif. Author manuscript; Available in PMC 2026 Jul 14. (PMC13364205; doi:10.1016/j.paid.2025.113549)
Supplement: 1 [file NIHMS2188431-supplement-1.docx]

Supplement: The Effect of Personality on Probability of Contracting SARS-CoV-2 in the United States

## Data Analytic Approach and Preregistered Analyses

### Personality Trait Relationship with SARS-CoV-2 Infections

We assessed the zero-order relationships between traits and seropositivity, using a standard Pearson product-moment correlation (equivalent to the point-biserial in this case). Seropositivity was a dichotomous variable indicating whether the participant ever tested seropositive to SARS-CoV-2 during the study (1) or never (0). Participants were weighted in accordance with U.S. Census demographic categories, to best approximate representative effect sizes. We report Pearson’s *r* for bivariate associations and odds ratios (ORs) from logistic regression models. These metrics align with our preregistration, are standard in personality–epidemiology research, and facilitate comparison with prior work. While other effect size metrics (e.g., Cohen’s *d*) are mathematically related to *r* and OR, we chose to retain these formats to maintain interpretability and consistency. We also report both uncorrected *p-*values and Holm corrected *p*-values (Holm, 1979) for transparency.

### Personality Prediction of SARS-CoV-2 Infection Overall and Beyond Demographics

We fit binomial logistic multilevel models (MLM) with assessments (Level 1) nested within person (Level 2). The outcome was whether the participant tested seropositive from infection (i.e., not vaccination), measured separately at each assessment. Level 1 predictors were the estimated prevalence of SARS-CoV-2 in participants’ county of residence on the date of assessment. We used county-level SARS-CoV-2 prevalence as a covariate rather than state-level prevalence because county estimates provide finer-grained measures of local infection risk. This approach better captures the variation in potential exposure environments experienced by participants and reduces the ecological imprecision inherent in state-level aggregates.

Level 2 predictors were person-level personality traits and demographics (age, sex, race, ethnicity, urban/rural residence, and education). While many covariates are objectively not caused by personality (e.g., age), education has a complex bi-directional causal relationship with traits, notably openness, that unfolds over the lifespan (Furnham & Cheng, 2016). We chose to include education here as a covariate because it is plausibly a common cause of both mid- and late-life openness and infection risk, in part through its association with health literacy and related behaviors. More specifically, given that personality was measured in adulthood after most participants’ formal education was completed, education is unlikely to operate as a mediator or collider in this context.

One model was fit for each trait and intercepts were allowed to vary across participants. We provide odds ratios to aid interpretation. We did not include all Big Five traits in a single model because of substantial intercorrelations among traits, which can lead to multicollinearity and unstable parameter estimates. Modeling each trait separately provides more stable and interpretable estimates of its overall association with infection risk.

We examined the intraclass correlation coefficient (ICC) for seropositivity at the U.S. state level. The observed ICC was approximately .00, indicating that none of the variance in seropositivity was attributable to differences between states. This value is consistent with prior work showing minimal personality-related variance between states (Elleman et al., 2018) and supports the decision not to model state-level clustering in our primary analyses.

### Personality Trait Prediction of Change in Risk Over Time

Longer evasion of infection may be influenced by personality or interaction with the changing nature of the pandemic. Including time as a categorical variable accounts for variation in infection risk across distinct data collection periods, defined by substantial changes in local and national pandemic conditions (e.g., new variants, public health policy shifts, changes in vaccination coverage). We tested whether personality traits predicted change in risk over time, modifying the multilevel models (section 2.4.2) by including time as a predictor at Level 1, with time operationalized as a categorical variable in discrete three-month blocks (see Supplement section 1.9 for detail). This allowed control for period-specific baseline differences in exposure likelihood and testing of whether personality–infection associations were consistent across these epidemiological contexts, rather than confounded by temporal shifts in infection dynamics.

## Blood Tests and Assessment of Seropositivity

The majority of participants gave 80 μl blood samples using a Mitra microsampling kit, drawing blood from the fingertip, and sent the sample by mail. A small number of participants also provided 18ml of blood through venipuncture. Blood samples underwent serological assay for antibodies using enzyme-linked immunoassay (ELISA). To determine the threshold measurements for seropositivity, 56 true positive and 300 true negative blood samples were assayed, and seropositivity was defined as mean optical density plus three standard deviations as previously described (Kalish et al., 2021; Klumpp-Thomas et al., 2021). Categorization of seropositivity required both spike protein positivity and receptor binding domain (RBD) positivity, for either IgG or IgM, with IgA providing additional discrimination, but without being sufficient on its own to categorize a sample as seropositive. Specificity was 100% (95% CI [96.4%, 100%]) and sensitivity was 100% (95% CI [76.8%, 100%]) (Klumpp-Thomas et al., 2021). Importantly, serological testing can distinguish between a COVID-19 vaccine immune system response and SARS-CoV-2 immune system response, as SARS-CoV-2 infection produces antibodies to the nucleocapsid protein, whereas the vaccine does not. The primary outcome was seropositivity, indicating SARS-CoV-2 infection. Antibody testing was the most appropriate approach, given our interest in who had been infected over the preceding six months, not just the narrow window of current infection (viral testing). For further detail on blood sampling and serological assay procedures, see Kalish et al. (2021) and Klumpp-Thomas et al. (2021).

## Descriptive Statistics for Scales Measuring Individual Differences

**Table S1**

*Descriptive Statistics for Scales Measuring Individual Differences*

| Personality Trait | Mean | SD | Min | Max | Range | SE |
| --- | --- | --- | --- | --- | --- | --- |
| Extraversion | 3.61 | 0.47 | 1.40 | 4.95 | 3.55 | 0.01 |
| Assertiveness | 3.58 | 0.56 | 1.40 | 5.00 | 3.60 | 0.01 |
| Enthusiasm | 3.64 | 0.57 | 1.10 | 5.00 | 3.90 | 0.01 |
| Agreeableness | 4.04 | 0.36 | 1.75 | 5.00 | 3.25 | 0.00 |
| Compassion | 4.11 | 0.43 | 1.70 | 5.00 | 3.30 | 0.01 |
| Politeness | 3.98 | 0.46 | 1.30 | 5.00 | 3.70 | 0.01 |
| Conscientiousness | 3.66 | 0.46 | 1.50 | 5.00 | 3.50 | 0.01 |
| Industriousness | 3.63 | 0.54 | 1.40 | 5.00 | 3.60 | 0.01 |
| Orderliness | 3.68 | 0.55 | 1.30 | 5.00 | 3.70 | 0.01 |
| Neuroticism | 2.41 | 0.56 | 1.00 | 4.80 | 3.80 | 0.01 |
| Volatility | 2.39 | 0.62 | 1.00 | 5.00 | 4.00 | 0.01 |
| Withdrawal | 2.42 | 0.61 | 1.00 | 4.90 | 3.90 | 0.01 |
| Openness/Intellect | 3.94 | 0.37 | 2.30 | 5.00 | 2.70 | 0.00 |
| Intellect | 4.03 | 0.46 | 1.60 | 5.00 | 3.40 | 0.01 |
| Openness | 3.86 | 0.48 | 1.70 | 5.00 | 3.30 | 0.01 |
| General Risk Perception | 4.70 | 0.58 | 1.57 | 6.57 | 5.00 | 0.01 |
| Ethical | 5.61 | 0.83 | 1.00 | 7.00 | 6.00 | 0.01 |
| Financial | 5.05 | 0.81 | 1.00 | 7.00 | 6.00 | 0.01 |
| Health | 5.52 | 0.86 | 1.00 | 7.00 | 6.00 | 0.01 |
| Recreational | 4.70 | 1.02 | 1.00 | 7.00 | 6.00 | 0.01 |
| Social | 2.60 | 0.75 | 1.00 | 6.50 | 5.50 | 0.01 |
| General Risk Taking | 2.78 | 0.60 | 1.07 | 5.47 | 4.40 | 0.01 |
| Ethical | 1.61 | 0.67 | 1.00 | 5.33 | 4.33 | 0.01 |
| Financial | 2.31 | 0.80 | 1.00 | 7.00 | 6.00 | 0.01 |
| Health | 2.10 | 0.93 | 1.00 | 7.00 | 6.00 | 0.01 |
| Recreational | 2.71 | 1.25 | 1.00 | 7.00 | 6.00 | 0.02 |
| Social | 5.15 | 0.92 | 1.17 | 7.00 | 5.83 | 0.01 |
| Optimism | 3.75 | 0.50 | 1.60 | 5.00 | 3.40 | 0.01 |

*Note.* Big Five personality (*n* = 4,739), risk perception (*n* = 4,731), risk taking (*n* = 4,721), optimism (n = 4,738).

## Seropositive by Sex

Table S2 shows the numbers and percentages of females and males who were seropositive for the first time at each assessment time period. A greater proportion of females than males were seropositive at the first two assessment time periods, but a slightly lower proportion of females were seropositive at assessment time 3.

**Table S2**

*First Time Seropositivity by Participant Sex*

| Characteristic | Female n = 3,250 | Male n = 2,908 |
| --- | --- | --- |
| Ever Positive | 1,720 (53%) | 1,306 (45%) |
| When Positive |  |  |
| Month 00 | 310 (9.5%) | 203 (7.0%) |
| Month 06 | 1,187 (37%) | 890 (31%) |
| Month 12 | 223 (6.9%) | 213 (7.3%) |
| Never positive | 1,530 (47%) | 1,602 (55%) |

*Note.* n (%). *N* = 6,158, including 1,419 participants who did not complete any personality questionnaires.

## Correlations between personality traits and covariates

Correlations between personality traits and covariates are shown in Table S3. Consistent with previous research, males were likely to be lower on agreeableness (*r* = -.25, *p* < .001) and higher on risk taking (*r* = .21, *p* < .001). Those higher on risk taking were likely to be younger (*r* = -.20 with age, *p* < .001), and risk taking was negatively correlated with risk perception (*r* = -.67, *p* < .001).

**Table S3**

*Correlations Between Personality Variables, Demographics, and Covariates*

|  | 1 | 2 | 3 | 4 | 5 | 6 | 7 | 8 | 9 | 10 | 11 | 12 | 13 | 14 | 15 | 16 | 17 | 18 |
| --- | --- | --- | --- | --- | --- | --- | --- | --- | --- | --- | --- | --- | --- | --- | --- | --- | --- | --- |
| 1. Extraversion |  |  |  |  |  |  |  |  |  |  |  |  |  |  |  |  |  |  |
| 2. Agreeableness | .12 |  |  |  |  |  |  |  |  |  |  |  |  |  |  |  |  |  |
| 3. Conscientiousness | .32 | .11 |  |  |  |  |  |  |  |  |  |  |  |  |  |  |  |  |
| 4. Neuroticism | -.32 | -.15 | -.32 |  |  |  |  |  |  |  |  |  |  |  |  |  |  |  |
| 5. Openness/Intellect | .37 | .20 | .08 | -.18 |  |  |  |  |  |  |  |  |  |  |  |  |  |  |
| 6. Risk Perception | .02 | .33 | .18 | .06 | -.04 |  |  |  |  |  |  |  |  |  |  |  |  |  |
| 7. Risk Taking | .01 | -.33 | -.22 | .06 | .08 | -.67 |  |  |  |  |  |  |  |  |  |  |  |  |
| 8. Optimism | .46 | .18 | .30 | -.68 | .23 | -.03 | -.03 |  |  |  |  |  |  |  |  |  |  |  |
| 9. Age | -.01 | .09 | .03 | -.23 | -.02 | .18 | -.20 | .16 |  |  |  |  |  |  |  |  |  |  |
| 10. Sex (Male) | -.04 | -.25 | -.06 | -.08 | .03 | -.27 | .21 | .01 | -.03 |  |  |  |  |  |  |  |  |  |
| 11. Urban vs Rural | -.03 | -.03 | -.01 | .02 | -.01 | -.02 | .04 | -.03 | .06 | .01 |  |  |  |  |  |  |  |  |
| 12. American Indian / Alaska Native vs White Only | .00 | .00 | -.03 | .00 | .02 | .01 | .03 | -.01 | -.04 | -.02 | .01 |  |  |  |  |  |  |  |
| 13. Asian Only vs White Only | -.03 | -.01 | .01 | .03 | -.04 | .02 | -.01 | -.04 | -.10 | -.03 | -.06 | -.04 |  |  |  |  |  |  |
| 14. Black Only vs White Only | .02 | .05 | .02 | -.02 | .02 | .05 | -.03 | .01 | -.09 | -.10 | -.07 | -.05 | -.07 |  |  |  |  |  |
| 15. Multiple Races vs White Only | .00 | -.02 | -.03 | .03 | .02 | -.02 | .02 | -.05 | -.07 | -.01 | -.02 | -.02 | -.04 | -.04 |  |  |  |  |
| 16. Other vs White Only | .01 | .01 | .01 | .01 | .01 | .01 | .00 | .01 | -.08 | .00 | -.03 | -.02 | -.03 | -.04 | -.02 |  |  |  |
| 17. Ethnicity Hispanic | .05 | -.03 | .02 | .02 | .02 | .02 | .01 | .01 | -.20 | .00 | -.08 | .10 | -.09 | -.07 | .04 | .23 |  |  |
| 18. Education (≤ High School) | .00 | .00 | .01 | .00 | -.05 | .01 | -.03 | -.02 | -.01 | .02 | .04 | .00 | -.02 | -.03 | -.01 | .01 | .02 |  |
| 19. Education (College) | -.01 | -.01 | .00 | .02 | -.07 | .02 | .00 | -.04 | .03 | .01 | .05 | .03 | -.06 | .00 | .01 | .01 | .03 | -.07 |

*Note.* *N* = 6,158, except Big Five personality (*n* = 4,739), risk perception (*n* = 4,731), risk taking (*n* = 4,721), optimism (n = 4,738). Categorical variables are dummy coded, with most populated group serving as the reference group (Women, Urban dwellers, White only, Non-Hispanic, and College degree or more educated). Significance levels are: *p* < 0.05 (*r* ≥ |.025|), *p* < .01 (*r* ≥ |.033|), *p* < .001 (*r* ≥ |.042|), except for correlations including Big Five personality, risk perception, risk taking, and optimism: *p* < 0.05 (*r* ≥ |.029|), *p* < .01 (*r* ≥ |.038|), *p* < .001 (*r* ≥ |.048|).

## To what extent can personality predict SARS-CoV-2 infection above and beyond demographic factors?

After controlling for demographic factors, we found that no personality traits emerged as predictive of SARS-CoV-2 infection after Holm (1979) correction (Table S4).

**Table S4**

*Relationship of Personality Traits to SARS-CoV-2 Seropositivity Above and Beyond Demographic Control Variables and COVID-19 Prevalence in County of Residence*

|  | Unstandardized Effect | | | | Standardized Effect | | | p-value | |
| --- | --- | --- | --- | --- | --- | --- | --- | --- | --- |
| Personality Trait | Estimate | SE | OR | Estimate | | SE | OR | Raw | Corrected |
| Extraversion | -0.18 | 0.52 | 0.83 | -0.19 | | 0.54 | 0.83 | .730 | > .999 |
| Assertiveness | -0.10 | 0.45 | 0.90 | -0.12 | | 0.54 | 0.88 | .818 | > .999 |
| Enthusiasm | -0.02 | 0.41 | 0.98 | -0.03 | | 0.50 | 0.97 | .954 | > .999 |
| Agreeableness | 1.05 | 0.66 | 2.86 | 0.82 | | 0.51 | 2.27 | .108 | > .999 |
| Compassion | 0.43 | 0.60 | 1.54 | 0.40 | | 0.55 | 1.49 | .470 | > .999 |
| Politeness | 0.84 | 0.51 | 2.31 | 0.83 | | 0.50 | 2.29 | .100 | > .999 |
| Conscientiousness | 0.32 | 0.50 | 1.38 | 0.32 | | 0.50 | 1.38 | .521 | > .999 |
| Industriousness | -0.24 | 0.46 | 0.79 | -0.28 | | 0.54 | 0.76 | .603 | > .999 |
| Orderliness | 0.74 | 0.42 | 2.09 | 0.87 | | 0.50 | 2.40 | .080 | > .999 |
| Neuroticism | 0.43 | 0.43 | 1.53 | 0.51 | | 0.51 | 1.67 | .315 | > .999 |
| Volatility | 0.23 | 0.37 | 1.26 | 0.31 | | 0.50 | 1.36 | .535 | > .999 |
| Withdrawal | 0.50 | 0.43 | 1.65 | 0.65 | | 0.56 | 1.92 | .243 | > .999 |
| Openness/Intellect | -1.74 | 0.66 | 0.18 | -1.41 | | 0.54 | 0.24 | .008 | .235 |
| Intellect | -1.17 | 0.53 | 0.31 | -1.18 | | 0.53 | 0.31 | .027 | .695 |
| Openness | -0.99 | 0.51 | 0.37 | -1.03 | | 0.53 | 0.36 | .052 | > .999 |
| General Risk Perception | 0.99 | 0.43 | 2.69 | 1.25 | | 0.54 | 3.47 | .021 | .565 |
| Ethical | 0.59 | 0.29 | 1.80 | 1.05 | | 0.51 | 2.86 | .040 | .994 |
| Financial | 0.38 | 0.30 | 1.46 | 0.66 | | 0.51 | 1.94 | .197 | > .999 |
| Health | 0.26 | 0.28 | 1.30 | 0.49 | | 0.53 | 1.63 | .355 | > .999 |
| Recreational | 0.24 | 0.25 | 1.27 | 0.53 | | 0.56 | 1.70 | .343 | > .999 |
| Social | 0.38 | 0.33 | 1.46 | 0.62 | | 0.53 | 1.85 | .247 | > .999 |
| General Risk Taking | -0.46 | 0.43 | 0.63 | -0.59 | | 0.56 | 0.55 | .287 | > .999 |
| Ethical | 0.20 | 0.35 | 1.22 | 0.28 | | 0.50 | 1.32 | .578 | > .999 |
| Financial | -0.49 | 0.30 | 0.61 | -0.83 | | 0.51 | 0.43 | .102 | > .999 |
| Health | 0.00 | 0.27 | 1.00 | 0.00 | | 0.54 | 1.00 | .996 | > .999 |
| Recreational | -0.11 | 0.21 | 0.90 | -0.30 | | 0.57 | 0.74 | .601 | > .999 |
| Social | -0.41 | 0.27 | 0.67 | -0.81 | | 0.54 | 0.45 | .133 | > .999 |
| Optimism | -0.44 | 0.52 | 0.64 | -0.48 | | 0.55 | 0.62 | .390 | > .999 |

*Note*. Big Five personality (*n* = 4,739), risk perception (*n* = 4,731), risk taking (*n* = 4,721), optimism (n = 4,738).

SE = Standard Error, OR = Odds Ratio. Corrected *p*-values result from a Holm (1979) correction.

## Relationship Between Personality and Seropositivity Above and Beyond Demographics, Omitting County COVID-19 Prevalence

**Table S5**

*Relationship of Personality Traits to SARS-CoV-2 Seropositivity Above and Beyond Demographic Control Variables, Omitting COVID-19 Prevalence in County of Residence*

|  | Unstandardized Effect | | | | Standardized Effect | | | p-value | |
| --- | --- | --- | --- | --- | --- | --- | --- | --- | --- |
| Personality Trait | Estimate | SE | OR | Estimate | | SE | OR | Raw | Corrected |
| Extraversion | -0.03 | 0.05 | 0.97 | -0.04 | | 0.05 | 0.97 | .504 | > .999 |
| Assertiveness | -0.04 | 0.04 | 0.96 | -0.05 | | 0.05 | 0.95 | .333 | > .999 |
| Enthusiasm | -0.01 | 0.04 | 0.99 | -0.01 | | 0.05 | 0.99 | .870 | > .999 |
| Agreeableness | 0.12 | 0.07 | 1.13 | 0.10 | | 0.05 | 1.10 | .082 | > .999 |
| Compassion | 0.10 | 0.06 | 1.11 | 0.10 | | 0.05 | 1.10 | .079 | > .999 |
| Politeness | 0.06 | 0.06 | 1.06 | 0.06 | | 0.05 | 1.06 | .276 | > .999 |
| Conscientiousness | 0.01 | 0.05 | 1.01 | 0.01 | | 0.05 | 1.01 | .788 | > .999 |
| Industriousness | -0.05 | 0.05 | 0.95 | -0.06 | | 0.05 | 0.95 | .299 | > .999 |
| Orderliness | 0.07 | 0.05 | 1.07 | 0.08 | | 0.05 | 1.08 | .140 | > .999 |
| Neuroticism | 0.05 | 0.05 | 1.05 | 0.06 | | 0.05 | 1.06 | .256 | > .999 |
| Volatility | 0.03 | 0.04 | 1.03 | 0.04 | | 0.05 | 1.04 | .433 | > .999 |
| Withdrawal | 0.05 | 0.04 | 1.06 | 0.07 | | 0.05 | 1.07 | .203 | > .999 |
| Openness/Intellect | **-0.21*** | 0.07 | 0.81 | **-0.17*** | | 0.05 | 0.85 | .002 | .050 |
| Intellect | -0.13 | 0.05 | 0.88 | -0.13 | | 0.05 | 0.88 | .019 | .489 |
| Openness | -0.13 | 0.05 | 0.87 | -0.14 | | 0.05 | 0.87 | .009 | .234 |
| General Risk Perception | 0.08 | 0.05 | 1.08 | 0.10 | | 0.06 | 1.10 | .088 | > .999 |
| Ethical | 0.05 | 0.03 | 1.05 | 0.09 | | 0.05 | 1.10 | .096 | > .999 |
| Financial | 0.03 | 0.03 | 1.03 | 0.05 | | 0.05 | 1.05 | .370 | > .999 |
| Health | 0.03 | 0.03 | 1.03 | 0.06 | | 0.06 | 1.06 | .324 | > .999 |
| Recreational | 0.02 | 0.03 | 1.02 | 0.05 | | 0.06 | 1.05 | .403 | > .999 |
| Social | 0.04 | 0.03 | 1.05 | 0.07 | | 0.05 | 1.07 | .182 | > .999 |
| General Risk Taking | -0.02 | 0.04 | 0.98 | -0.03 | | 0.06 | 0.97 | .620 | > .999 |
| Ethical | 0.07 | 0.04 | 1.08 | 0.10 | | 0.05 | 1.11 | .057 | > .999 |
| Financial | -0.04 | 0.03 | 0.96 | -0.07 | | 0.05 | 0.93 | .179 | > .999 |
| Health | 0.01 | 0.03 | 1.01 | 0.02 | | 0.06 | 1.02 | .661 | > .999 |
| Recreational | 0.00 | 0.02 | 1.00 | -0.01 | | 0.06 | 0.99 | .852 | > .999 |
| Social | -0.05 | 0.03 | 0.95 | -0.10 | | 0.05 | 0.90 | .057 | > .999 |
| Optimism | -0.02 | 0.05 | 0.98 | -0.02 | | 0.05 | 0.98 | .681 | > .999 |

*Note.* Big Five personality (*n* = 4,739), risk perception (*n* = 4,731), risk taking (*n* = 4,721), optimism (n = 4,738).

All significant correlations are bold: **p* < .05

## Personality Trait Relationship with SARS-CoV-2 Infections

Analysis of correlations between traits and seropositivity without demographic controls—testing seropositive from infection (not vaccination) at any point during the study (1) or never (0)—found 13 significant but very small correlations before Holm correction (Table 6 of main article). However, there were only three significant results after Holm correction, with agreeableness having a *positive* correlation with seropositivity (likely caused by the sex difference in seropositivity), and both the broad openness/intellect factor and narrower openness aspect having a negative correlation with seropositivity.

## Personality Moderation of Change in Seropositivity Over Time

We preregistered and tested whether personality traits predict change in risk over time—whether any traits moderate change in seropositivity risk over time. We modified the multilevel models used to assess whether personality can predict SARS-CoV-2 infection overall and above-and-beyond demographic factors, by including time as a predictor at Level 1. Time was operationalized as a categorical variable, with time “chunked” into discrete blocks. This allowed for estimation of nonlinear trends. The blocks were (1) April – June 2020, (2) July – September 2020, (3) October – December 2020, (4) January – March 2021, (5) April – June 2021, (6) July – September 2021 (see Table S6). The coefficient was allowed to vary across participant and be predicted from personality. One model was estimated for each personality trait. Here, we report the effect size (regression coefficient of the interaction), uncorrected p-value, and Holm-corrected p-value, applying the correction across the set of regression coefficients presented here. Table S7 shows the results of these analyses

*Planned sensitivity analysis.* Planned sensitivity analyses relating to effect of time interval on change in seropositivity over time were not pursued, given the trivial effects of time.

**Table S6**

*Counts and Seropositivity by Blocks*

| Block | *N* | Date Range | Seropositive | Missing Data |
| --- | --- | --- | --- | --- |
| Block 1 | 3,943 | 29 April 2020 to 30 June 2020 | 323 (8.4%) | 81 |
| Block 2 | 2,215 | 1 July 2020 to 10 Sep 2020 | 190 (8.9%) | 90 |
| Block 3 | 2,089 | 21 Oct 2020 to 31 Dec 2020 | 625 (34%) | 270 |
| Block 4 | 3,880 | 1 Jan 2021 to 30 March 2021 | 1,750 (54%) | 621 |
| Block 5 | 4,138 | 1 April 2021 to 30 June 2021 | 1,705 (51%) | 765 |
| Block 6 | 947 | 1 July 2021 to 16 Sep 2021 | 361 (53%) | 272 |

## *Note.* *N* = 6,158, including 1,419 participants who did not complete any personality questionnaires. Shows for each assessment the time period covered by that assessment and block each date is assigned to. Dates for blocks pre-specified during preregistration. Blocks are used in subsequent analyses, while assessment number is ignored. Shows number and percentage of total block testing positive. Percentages calculated from total seropositives out of available sample for that time period (excludes Missing Data participants, not sero-tested for that period).

**Table S7**

*Personality Trait Moderation of Change in SARS-CoV-2 Seropositivity Over Time: Estimated Slopes of Personality by Block of Time*

| Block | Estimate | Confidence Interval Low | Confidence Interval High | Odds Ratio | p*-v*alue |
| --- | --- | --- | --- | --- | --- |
| Extraversion |  |  |  |  |  |
| Block 1 | 0.02 | -1.33 | 1.38 | 1.02 | .972 |
| Block 2 | -0.16 | -2.24 | 1.92 | 0.85 | .881 |
| Block 3 | 0.06 | -0.98 | 1.11 | 1.06 | .907 |
| Block 4 | 0.05 | -0.90 | 1.00 | 1.05 | .918 |
| Block 5 | -0.09 | -1.05 | 0.88 | 0.92 | .859 |
| Block 6 | -0.03 | -1.45 | 1.39 | 0.97 | .970 |
| Agreeableness |  |  |  |  |  |
| Block 1 | 0.04 | -1.25 | 1.33 | 1.04 | .948 |
| Block 2 | 0.28 | -2.03 | 2.58 | 1.32 | .815 |
| Block 3 | 0.13 | -0.89 | 1.15 | 1.14 | .802 |
| Block 4 | 0.45 | -0.48 | 1.39 | 1.58 | .342 |
| Block 5 | 0.23 | -0.72 | 1.18 | 1.26 | .633 |
| Block 6 | 0.57 | -0.68 | 1.81 | 1.76 | .371 |
| Conscientiousness |  |  |  |  |  |
| Block 1 | 0.10 | -1.25 | 1.46 | 1.11 | .880 |
| Block 2 | 0.04 | -2.21 | 2.28 | 1.04 | .974 |
| Block 3 | 0.09 | -0.94 | 1.13 | 1.10 | .858 |
| Block 4 | 0.14 | -0.81 | 1.09 | 1.15 | .776 |
| Block 5 | -0.01 | -0.97 | 0.95 | 0.99 | .981 |
| Block 6 | -0.12 | -1.54 | 1.29 | 0.88 | .865 |
| Neuroticism |  |  |  |  |  |
| Block 1 | -0.26 | -1.68 | 1.16 | 0.77 | .715 |
| Block 2 | 0.16 | -2.07 | 2.38 | 1.17 | .889 |
| Block 3 | -0.33 | -1.41 | 0.76 | 0.72 | .555 |
| Block 4 | 0.13 | -0.85 | 1.10 | 1.14 | .798 |
| Block 5 | 0.09 | -0.89 | 1.08 | 1.10 | .851 |
| Block 6 | -0.15 | -1.59 | 1.29 | 0.86 | .840 |
| Openness/Intellect |  |  |  |  |  |
| Block 1 | 0.08 | -1.28 | 1.43 | 1.08 | .913 |
| Block 2 | -0.16 | -2.35 | 2.03 | 0.85 | .886 |
| Block 3 | -0.07 | -1.09 | 0.95 | 0.93 | .896 |
| Block 4 | -0.02 | -0.96 | 0.93 | 0.98 | .971 |
| Block 5 | -0.15 | -1.10 | 0.81 | 0.86 | .761 |
| Block 6 | -0.41 | -1.85 | 1.04 | 0.67 | .580 |
| General Risk Perception |  |  |  |  |  |
| Block 1 | 0.13 | -1.32 | 1.58 | 1.14 | .862 |
| Block 2 | -0.14 | -2.18 | 1.90 | 0.87 | .893 |
| Block 3 | 0.05 | -1.06 | 1.15 | 1.05 | .931 |
| Block 4 | 0.36 | -0.63 | 1.36 | 1.44 | .475 |
| Block 5 | 0.11 | -0.90 | 1.13 | 1.12 | .824 |
| Block 6 | -0.02 | -1.36 | 1.32 | 0.98 | .977 |
| General Risk Taking |  |  |  |  |  |
| Block 1 | -0.20 | -1.67 | 1.27 | 0.82 | .793 |
| Block 2 | -0.07 | -2.25 | 2.11 | 0.93 | .951 |
| Block 3 | -0.11 | -1.22 | 1.00 | 0.89 | .844 |
| Block 4 | -0.22 | -1.22 | 0.78 | 0.80 | .666 |
| Block 5 | -0.03 | -1.04 | 0.99 | 0.97 | .960 |
| Block 6 | -0.10 | -1.48 | 1.29 | 0.91 | .888 |
| Optimism |  |  |  |  |  |
| Block 1 | 0.35 | -1.05 | 1.75 | 1.42 | .622 |
| Block 2 | -0.18 | -2.35 | 1.98 | 0.83 | .868 |
| Block 3 | 0.40 | -0.69 | 1.49 | 1.49 | .469 |
| Block 4 | -0.08 | -1.05 | 0.89 | 0.92 | .874 |
| Block 5 | -0.08 | -1.06 | 0.90 | 0.92 | .873 |
| Block 6 | 0.19 | -1.23 | 1.61 | 1.21 | .795 |

*Note.* Big Five personality (*n* = 4,739), risk perception (*n* = 4,731), risk taking (*n* = 4,721), optimism (n = 4,738).

## Mediation Analyses

We assessed the degree to which personality traits may influence seropositivity through vaccination. These preregistered analyses and results are shown in Table S8. During the data collection period, COVID-19 vaccines were developed and distributed in the United States. At blood collection time points 2 and 3, participants self-reported whether they had been vaccinated or not. Given associations between personality traits and other health behaviors—notably, medication adherence (Hill & Roberts, 2011)—we expected that personality traits would be associated with vaccination, and that this would, in turn, mediate the relationship between personality and seropositivity across the study. Regarding non-COVID vaccines, risk-perception has shown small associations with getting vaccinated (Brewer et al., 2007), and positive attitudes and intentions to vaccinate have been associated with higher agreeableness, conscientiousness, and extraversion (Bleidhorn et al., 2025; Browne et al., 2015; Lin & Wang, 2020). Regarding COVID vaccines, *attitudes* to vaccination were more positive if higher on agreeableness and openness (Bleidhorn et al., 2025). *Actual COVID vaccination* has been found to be associated with higher neuroticism and agreeableness, lower openness, and being politically liberal (Arumäe et al., 2024), but Bleidhorn et al. found no associations between actual vaccination and personality.

We therefore planned to perform a simple mediation analysis, in which personality was entered as the predictor variable, vaccination as the mediator variable, and whether the participant tested seropositive at either their second or third assessment. We restricted these analyses to only participants who were seronegative at the first assessment. We fitted this model for each trait (each of the Big Five and each of the aspects, each of the DOSPERT domains, overall risk perception, overall risk-taking, and optimism). Direct, indirect, and total effects were calculated using bootstrapping (with 10,000 iterations); we report the median estimate and 95% confidence interval using the bias-corrected approach.

The outcome variable (Y) in these models is whether the participant ever tested positive for COVID-19. The predictor (X) was personality trait (one model per trait). The mediating variable (M) is whether the participant was ever vaccinated for COVID-19. Because both the mediating and outcome variables are binary, we used binary logistic regression models and bootstrap coefficient estimates (*N* = 100) to determine statistical significance.

For all models, we report 4 coefficients:

- *Indirect Effect*: The path from personality to seropositivity through vaccine. This represents the effect of personality on risk of infection through vaccines.
- *Direct Effect*: The path from personality to seropositivity not through vaccine. This represents the effect of personality on risk of infection through any mechanism that is not vaccines.
- *Total Effect*: The path from personality to seropositivity. This is identical to what is estimated via a zero-order correlation, although here we present unstandardized regression coefficients. This is also equivalent to the sum of the indirect and direct effects.
- *Proportion of Total*: This communicates the indirect effect as a proportion of the total effect. In other words, how much of the relationship between personality and seropositivity is through vaccination?

The main finding was that these effects were small and questionable—there were indirect effects of *p* < .05 for the factors of extraversion and agreeableness, aspects of assertiveness, compassion, and openness, and the health subscales of risk perception and risk taking. However, these effects were without controlling for demographics and for multiple tests, so are likely not robust. This makes sense given that personality had only weak associations with seropositivity to begin with, so there was not much total effect for personality to capture.

**Table S8**

*Mediation Analysis*

|  | Indirect Effect | | | | Direct Effect | | | | Total Effect | | | | Proportion of Total Effect | | | |
| --- | --- | --- | --- | --- | --- | --- | --- | --- | --- | --- | --- | --- | --- | --- | --- | --- |
|  |  | CI | |  |  | CI | |  |  | CI | |  |  | CI | |  |
| Personality Trait | b | LB | UB | *p* | b | LB | UB | *p* | b | LB | UB | *p* | b | LB | UB | *p* |
| Extraversion | 0.00 | 0.00 | 0.00 | .06 | -0.01 | -0.04 | 0.02 | .44 | -0.01 | -0.04 | 0.02 | .38 | 0.17 | -9.11 | 1.10 | .44 |
| Assertiveness | 0.00 | 0.00 | 0.00 | .02 | -0.01 | -0.03 | 0.01 | .42 | -0.01 | -0.03 | 0.01 | .32 | 0.14 | -3.12 | 2.16 | .34 |
| Enthusiasm | 0.00 | 0.00 | 0.00 | .04 | 0.00 | -0.02 | 0.02 | .84 | 0.00 | -0.02 | 0.02 | .74 | 0.28 | -0.70 | 2.90 | .74 |
| Agreeableness | 0.00 | 0.00 | 0.00 | .04 | 0.04 | 0.01 | 0.07 | < .001 | 0.04 | 0.01 | 0.08 | < .001 | 0.03 | 0.01 | 0.17 | .04 |
| Compassion | 0.00 | 0.00 | 0.00 | .06 | 0.04 | 0.01 | 0.07 | .02 | 0.04 | 0.01 | 0.07 | .02 | 0.04 | 0.00 | 0.15 | .08 |
| Politeness | 0.00 | 0.00 | 0.00 | .18 | 0.02 | -0.01 | 0.05 | .14 | 0.02 | -0.01 | 0.05 | .14 | 0.02 | -0.10 | 0.10 | .32 |
| Conscientiousness | 0.00 | 0.00 | 0.00 | .88 | 0.00 | -0.02 | 0.02 | .94 | 0.00 | -0.02 | 0.02 | .92 | -0.07 | -0.17 | 0.36 | .92 |
| Industriousness | 0.00 | 0.00 | 0.00 | .08 | -0.01 | -0.03 | 0.03 | .56 | -0.01 | -0.03 | 0.03 | .58 | -0.10 | -0.39 | 0.27 | .54 |
| Orderliness | 0.00 | 0.00 | 0.00 | < .001 | 0.01 | -0.01 | 0.03 | .40 | 0.01 | -0.01 | 0.03 | .46 | -0.09 | -1.12 | 0.83 | .46 |
| Neuroticism | 0.00 | 0.00 | 0.00 | .28 | 0.02 | -0.01 | 0.04 | .16 | 0.02 | -0.01 | 0.04 | .18 | -0.03 | -0.40 | 0.09 | .42 |
| Volatility | 0.00 | 0.00 | 0.00 | .22 | 0.01 | 0.00 | 0.03 | .12 | 0.01 | 0.00 | 0.03 | .12 | -0.02 | -0.20 | 0.16 | .30 |
| Withdrawal | 0.00 | 0.00 | 0.00 | .40 | 0.01 | -0.01 | 0.03 | .24 | 0.01 | -0.01 | 0.03 | .24 | -0.03 | -0.58 | 0.21 | .60 |
| Openness/Intellect | 0.00 | 0.00 | 0.00 | .24 | -0.06 | -0.09 | -0.01 | < .001 | -0.06 | -0.09 | -0.01 | < .001 | 0.01 | 0.00 | 0.06 | .24 |
| Intellect | 0.00 | 0.00 | 0.00 | .50 | -0.03 | -0.06 | -0.01 | < .001 | -0.03 | -0.06 | -0.01 | < .001 | -0.01 | -0.05 | 0.02 | .50 |
| Openness | 0.00 | 0.00 | 0.00 | .04 | -0.04 | -0.05 | -0.01 | .02 | -0.04 | -0.05 | -0.01 | .02 | 0.02 | 0.00 | 0.09 | .06 |
| General Risk Perception | 0.00 | 0.00 | 0.00 | .10 | 0.03 | 0.01 | 0.05 | < .001 | 0.03 | 0.01 | 0.05 | < .001 | 0.01 | 0.00 | 0.07 | .10 |
| Ethical | 0.00 | 0.00 | 0.00 | .64 | 0.02 | 0.00 | 0.04 | < .001 | 0.02 | 0.00 | 0.03 | < .001 | 0.00 | -0.04 | 0.02 | .64 |
| Financial | 0.00 | 0.00 | 0.00 | .24 | 0.01 | 0.00 | 0.03 | .12 | 0.01 | 0.00 | 0.03 | .12 | 0.02 | -0.08 | 0.11 | .36 |
| Health | 0.00 | 0.00 | 0.00 | .02 | 0.02 | 0.00 | 0.03 | .04 | 0.02 | 0.00 | 0.03 | .04 | 0.03 | 0.00 | 0.12 | .06 |
| Recreational | 0.00 | 0.00 | 0.00 | .50 | 0.01 | 0.00 | 0.03 | .02 | 0.01 | 0.00 | 0.03 | .02 | 0.01 | -0.03 | 0.07 | .52 |
| Social | 0.00 | 0.00 | 0.00 | .50 | 0.02 | 0.00 | 0.03 | .06 | 0.02 | 0.00 | 0.03 | .06 | 0.01 | -0.02 | 0.08 | .52 |
| General Risk Taking | 0.00 | 0.00 | 0.00 | .22 | -0.01 | -0.03 | 0.01 | .58 | -0.01 | -0.03 | 0.01 | .56 | 0.04 | -0.57 | 0.52 | .70 |
| Ethical | 0.00 | 0.00 | 0.00 | .64 | 0.01 | 0.00 | 0.03 | .14 | 0.01 | 0.00 | 0.03 | .14 | 0.00 | -0.13 | 0.09 | .74 |
| Financial | 0.00 | 0.00 | 0.00 | .48 | -0.01 | -0.02 | 0.00 | .14 | -0.01 | -0.02 | 0.00 | .14 | 0.01 | -0.18 | 0.10 | .62 |
| Health | 0.00 | 0.00 | 0.00 | .10 | 0.00 | -0.01 | 0.01 | .64 | 0.00 | -0.01 | 0.01 | .62 | 0.10 | -0.32 | 0.63 | .68 |
| Recreational | 0.00 | 0.00 | 0.00 | .66 | 0.00 | -0.01 | 0.00 | .60 | 0.00 | -0.01 | 0.00 | .60 | -0.02 | -0.33 | 0.23 | .74 |
| Social | 0.00 | 0.00 | 0.00 | .04 | -0.01 | -0.02 | 0.00 | .14 | -0.01 | -0.02 | 0.00 | .12 | 0.03 | -0.32 | 0.20 | .16 |
| Optimism | 0.00 | 0.00 | 0.00 | .96 | -0.01 | -0.04 | 0.02 | .50 | -0.01 | -0.04 | 0.02 | .50 | 0.00 | -1.35 | 0.17 | .86 |

*Note.* Big Five personality (*n* = 4,739), risk perception (*n* = 4,731), risk taking (*n* = 4,721), optimism (n = 4,738).

CI = confidence interval. LB = lower bound. UB = upper bound.

## Exploratory Survival Models

To estimate survival models, we restructured our data such that each participant had one row, and a column was included indicating that either the event (seropositivity) had occurred or it had not. For a baseline, we chose the earliest date of data collection in the sample. Some participants tested seropositive multiple times, and a small number tested seronegative after testing seropositive. For the purpose of these particular analyses, we considered a participant seropositive any time after testing positive and we recorded only the first date of testing positive. The baseline survival model is shown in Figure S1.

**Figure S1**

*Baseline Survival Model*


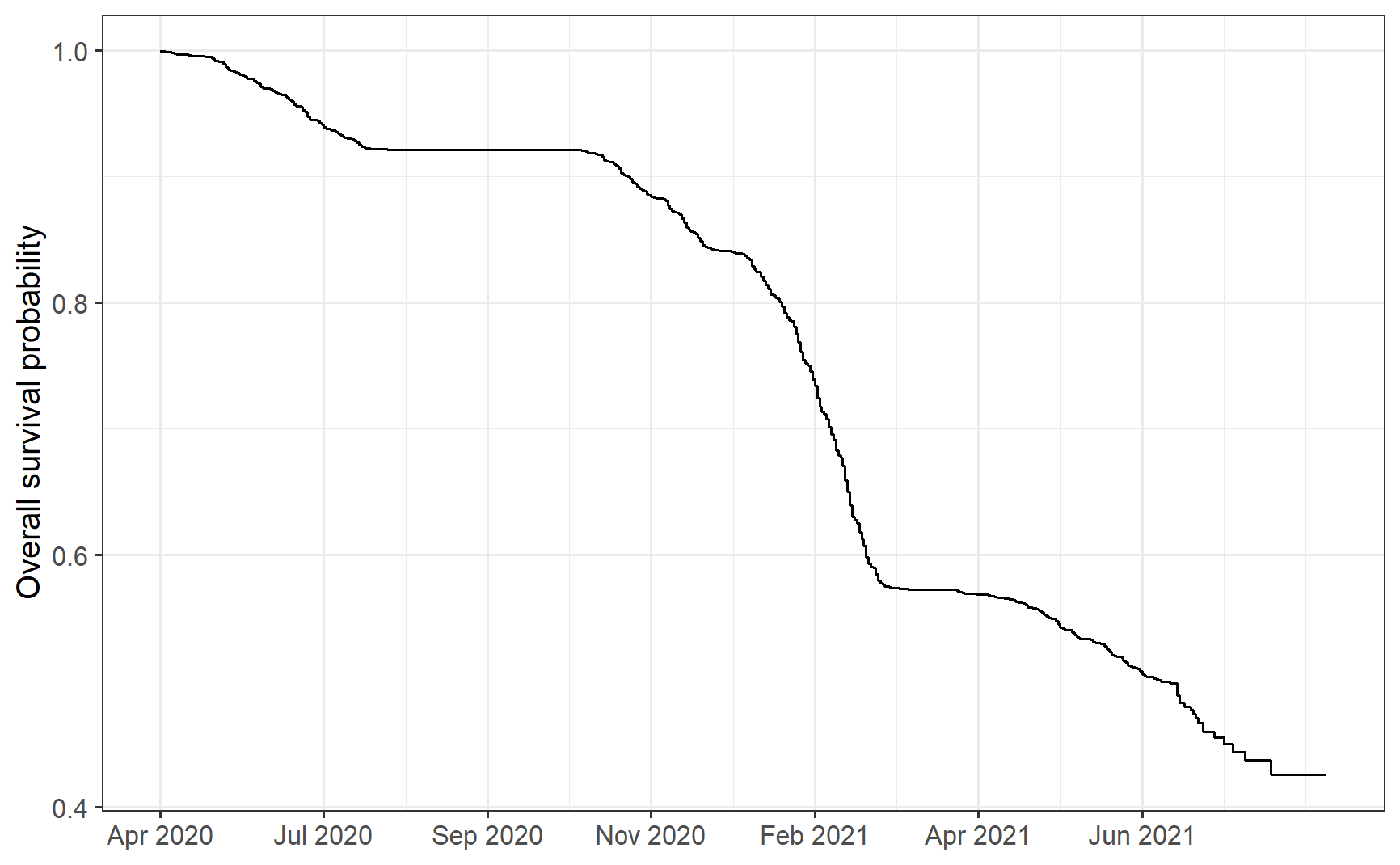


We fit Cox proportional hazards models to assess the relationship of the continuous personality variables to survival odds (Table S9).

**Table S9**

*Cox Proportional Hazards Models: Personality Only*

| Personality Trait | OR | SE | p-value | Lower CI | Upper CI |
| --- | --- | --- | --- | --- | --- |
| Extraversion | 0.98 | 0.04 | .694 | 0.91 | 1.07 |
| Assertiveness | 0.96 | 0.03 | .300 | 0.90 | 1.03 |
| Enthusiasm | 1.01 | 0.03 | .723 | 0.95 | 1.08 |
| Agreeableness | **1.12** | **0.05** | **.033** | **1.01** | **1.25** |
| Compassion | **1.11** | **0.05** | **.022** | **1.02** | **1.21** |
| Politeness | 1.05 | 0.04 | .232 | 0.97 | 1.14 |
| Conscientiousness | 1.03 | 0.04 | .465 | 0.95 | 1.12 |
| Industriousness | 0.99 | 0.04 | .842 | 0.93 | 1.06 |
| Orderliness | 1.05 | 0.04 | .156 | 0.98 | 1.13 |
| Neuroticism | 1.06 | 0.03 | .099 | 0.99 | 1.13 |
| Volatility | 1.04 | 0.03 | .213 | 0.98 | 1.10 |
| Withdrawal | 1.06 | 0.03 | .081 | 0.99 | 1.12 |
| Openness/Intellect | **0.85** | **0.05** | **.001** | **0.77** | **0.94** |
| Intellect | **0.92** | **0.04** | **.034** | **0.84** | **0.99** |
| Openness | **0.89** | **0.04** | **.003** | **0.82** | **0.96** |
| General Risk Perception | **1.10** | **0.03** | **.004** | **1.03** | **1.18** |
| Ethical | 1.04 | 0.02 | .075 | 1.00 | 1.09 |
| Financial | 1.05 | 0.02 | .062 | 1.00 | 1.10 |
| Health | **1.05** | **0.02** | **.039** | **1.00** | **1.10** |
| Recreational | **1.04** | **0.02** | **.026** | **1.01** | **1.08** |
| Social | 1.05 | 0.03 | .054 | 1.00 | 1.10 |
| General Risk Taking | 0.97 | 0.03 | .426 | 0.91 | 1.04 |
| Ethical | **1.07** | **0.03** | **.026** | **1.01** | **1.13** |
| Financial | 0.96 | 0.02 | .072 | 0.91 | 1.00 |
| Health | 1.00 | 0.02 | .845 | 0.96 | 1.05 |
| Recreational | 0.99 | 0.02 | .383 | 0.96 | 1.02 |
| Social | 0.97 | 0.02 | .102 | 0.93 | 1.01 |
| Optimism | 0.96 | 0.04 | .343 | 0.89 | 1.04 |

*Note.* Big Five personality (*n* = 4,739), risk perception (*n* = 4,731), risk taking (*n* = 4,721), optimism (n = 4,738).

OR= odds ratio. SE = standard error. CI = confidence interval. LB = lower bound. UB = upper bound. Significant correlations in bold.

Finally, we fit Cox proportional hazards models to assess the relationship of the continuous personality variables to survival odds, but controlling for our demographics of interest (Table S10).

**Table S10**

*Cox Proportional Hazards Models: Controlling for Demographics*

| Personality Trait | OR | SE | p-value | Lower CI | Upper CI |
| --- | --- | --- | --- | --- | --- |
| Extraversion | 0.97 | 0.04 | .451 | 0.89 | 1.05 |
| Assertiveness | 0.96 | 0.03 | .305 | 0.90 | 1.03 |
| Enthusiasm | 0.99 | 0.03 | .796 | 0.93 | 1.06 |
| Agreeableness | 1.04 | 0.06 | .496 | 0.93 | 1.16 |
| Compassion | 1.03 | 0.05 | .474 | 0.94 | 1.13 |
| Politeness | 1.02 | 0.04 | .697 | 0.93 | 1.11 |
| Conscientiousness | 1.01 | 0.04 | .865 | 0.93 | 1.10 |
| Industriousness | 0.99 | 0.04 | .869 | 0.93 | 1.07 |
| Orderliness | 1.02 | 0.04 | .654 | 0.95 | 1.09 |
| Neuroticism | 1.03 | 0.04 | .475 | 0.96 | 1.10 |
| Volatility | 1.02 | 0.03 | .450 | 0.96 | 1.09 |
| Withdrawal | 1.02 | 0.03 | .596 | 0.95 | 1.09 |
| Openness/Intellect | **0.84** | **0.05** | **.001** | **0.76** | **0.94** |
| Intellect | 0.93 | 0.04 | .094 | 0.86 | 1.01 |
| Openness | **0.87** | **0.04** | **< .001** | **0.81** | **0.94** |
| General Risk Perception | 1.04 | 0.04 | .234 | 0.97 | 1.12 |
| Ethical | 1.03 | 0.02 | .230 | 0.98 | 1.08 |
| Financial | 1.03 | 0.02 | .245 | 0.98 | 1.08 |
| Health | 1.01 | 0.02 | .771 | 0.96 | 1.06 |
| Recreational | 1.01 | 0.02 | .703 | 0.97 | 1.05 |
| Social | 1.03 | 0.03 | .306 | 0.98 | 1.08 |
| General Risk Taking | 1.01 | 0.03 | .697 | 0.95 | 1.08 |
| Ethical | **1.07** | **0.03** | **.018** | **1.01** | **1.13** |
| Financial | 0.98 | 0.03 | .450 | 0.93 | 1.03 |
| Health | 1.02 | 0.02 | .280 | 0.98 | 1.07 |
| Recreational | 1.01 | 0.02 | .510 | 0.98 | 1.04 |
| Social | 0.97 | 0.02 | .092 | 0.93 | 1.01 |
| Optimism | 0.97 | 0.04 | .467 | 0.90 | 1.05 |

*Note.* Big Five personality (*n* = 4,739), risk perception (*n* = 4,731), risk taking (*n* = 4,721), optimism (n = 4,738).

OR= odds ratio. SE = standard error. CI = confidence interval. LB = lower bound. UB = upper bound. Significant correlations in bold.

## Preregistration Deviations

Preregistration 1 was created 5 October 2020, and outlined our study hypotheses and general analytic plan before personality data collection began, but after the first of three tranches of blood tests were performed. We did not have access to any data at the time of preregistration 1.

Preregistration 2 was created 28 August 2021, after personality data collection had commenced, but before any data collation or access to data, which occurred after all three tranches of blood tests had been performed. Preregistration 2 was created to specify additional modeling details (e.g., inclusion of multilevel logistic models) based on the structure of the serological testing data.

Deviations from preregistration are detailed in Table S11, based on recommendations from Willroth and Atherton (2024).

**Table S11**

*Deviations from Preregistration and Unregistered Exploratory Additions*

| Deviations | | | | | |
| --- | --- | --- | --- | --- | --- |
| # | Details | | Original Wording | Deviation Description | Reader Impact |
| 1 | Type | Analysis | The original preregistration plan in preregistration 1 did not include an analysis of vaccination status mediation. | A mediation analysis, with vaccination status included as a mediator, was included in our preregistration 2 preregistered models. This analysis was added from preregistration 1, given vaccines were only known of and became available after pre-registration 1. | This deviation should not affect readers’ interpretation of the study or results. We adhered to the preregistered analysis of preregistration 2. |
|  | Reason | New knowledge |  |  |  |
|  | Timing | During data collection and before data access |  |  |  |
| 2 | Type | Analysis | Preregistration 2: Planned sensitivity analysis: The chunks described above may not be appropriately aligned to the seropositivity tests or may be too coarse/specific to identify the trend. We will additionally test different three-month chunks (e.g., March – May, February – April) as well as two-month and four-month chunks to see if conclusions differ. | Our preregistered analyses of personality moderation of change in seropositivity over time included planned sensitivity analysis. However, planned sensitivity analyses relating to effect of time interval on change in seropositivity over time were not pursued, given the trivial effects of time. | This deviation should not affect readers’ interpretation of the study or results, given the trivial effects of time in the analyses conducted. |
|  | Reason | New knowledge and redundancy |  |  |  |
|  | Timing | After results known |  |  |  |
| 3 | Type | Hypotheses | Preregistration 1: The hypotheses are that people will be more likely to test positive for SARS-CoV-2 antibodies if:  1. They are higher on the Extraversion factor.  2. They are lower on the Agreeableness factor.  3. They are lower on the Conscientiousness factor.  4. They are lower on Risk Perception.  5. They are higher on Risk Taking.  More specifically, in relation to the personality facets of the above factors, it is hypothesised that people will be more likely to test positive to SARS-CoV-2 antibodies if:  6. They are higher on the Enthusiasm facet of the Extraversion factor.  7. They are lower on the Compassion facet of the Agreeableness factor.  8. They are lower on the Politeness facet of the Agreeableness factor.  Preregistration 2: We will report the effect size of the correlation, the uncorrected *p-*value, and Holm corrected *p*-values (Holm, 1979). | Although not technically a deviation, our preregistration 2 specified analysing the uncorrected *p-*values, and Holm corrected *p*-values (Holm, 1979), however we were not precise in specifying which *p*-values would be used to test our hypotheses, the uncorrected *p*-values or the stricter, corrected *p*-values. | This deviation should not affect readers’ interpretation of the study or results, given we are transparent about which significant results are based on corrected versus uncorrected *p*-values. |
|  | Reason | Oversight of detail |  |  |  |
|  | Timing | After results known |  |  |  |
| 4 | Type | Analysis | Pre-registration 1: In addition to the hypotheses made, several exploratory personality variables will be evaluated:  1. Openness/Intellect  2. Neuroticism  3. Optimism  4. Worry about germs  Although the effect of these personality variables are uncertain and official hypotheses will not be made for them, estimates are that people will be more likely to test positive to SARS-CoV-2 antibodies if…  6. They were less worried about germs before the advent of COVID-19.  7. They are less worried about germs at the time of completing the questionnaire. | Pre-registration 1 listed exploratory analyses (not hypotheses) of the relationship between worry about germs (before and during the COVID pandemic) and infection. This was not pursued in pre-registration 2, given worry about germs consisted of only two items, which had not been previously used or psychometrically validated. | This deviation should not affect readers’ interpretation of the study or results. |
|  | Reason | Realization of weakness in method |  |  |  |
|  | Timing | During data collection and before data access |  |  |  |
| Unregistered Steps | | | | | |
| # | Details | | Original Wording | Unregistered Step Description | Reader Impact |
| 1 | Type | Analysis | Preregistration 2: We also propose the inclusion of key demographic factors: region of the United States, age group, sex, urban/rural, race, ethnicity, and education.  We will fit multilevel models (MLM) with assessments (Level 1) nested within person (Level 2). The outcome is seropositivity, measured separately at each assessment. Level 1 predictors will be the estimated prevalence of SARSCoV-2 in the participant’s county of residence on the date of assessment. Level 2 predictors are person-level demographic factors and personality. | In preregistration 2, analyses with demographic controls included county seroprevalence. However, we performed additional analyses with demographic controls but *not* including county seroprevalence. | This deviation may affect readers’ interpretation of these particular results, given these additional analyses were not originally hypothesized and were performed after results were known.  However, analyses are justified by the many asymptomatic and mild cases that never get detected, with underestimation likely varying by state and county, plus lack of knowledge about precise infection date. These issues were not foreseen when registering the analyses. |
|  | Timing | After results known |  |  |  |
| 2 | Type | Analysis | This issue and analyses were not addressed in the preregistration at all. | Analyses of Baseline and Cox proportional hazards models and survival models were conducted, to serve as sensitivity analyses to complement our primary logistic models. This was influenced by peer review. | This deviation should not affect readers’ interpretation of the study or results, apart from potentially providing additional confirmation of the role of trait openness in virus infection. |
|  | Timing | After results known |  |  |  |

# References

Arumäe, K., Realo, A., Ausmees, L., Allik, J., Esko, T., Fischer, K., et al. (2024). Self-and informant-reported personality traits and vaccination against COVID-19. *PLoS ONE*, *19*(3), e0287413. https://doi. org/10.1371/journal.pone.0287413

Bleidorn, W., Stahlmann, A. G., & Hopwood, C. J. (2025). Big Five personality traits and vaccination: A systematic review and meta-analysis. *Health Psychology, 44*(1), 44–56. <https://doi.org/10.1037/hea0001398>

Brewer, N. T., Chapman, G. B., Gibbons, F. X., Gerrard, M., McCaul, K. D., & Weinstein, N. D. (2007). Meta-analysis of the relationship between risk perception and health behavior: the example of vaccination. *Health psychology*, *26*(2), 136–145. https://doi.org/10.1037/0278-6133.26.2.136

Browne, M., Thomson, P., Rockloff, M. J., & Pennycook, G. (2015). Going against the herd: psychological and cultural factors underlying the ‘vaccination confidence gap’. *PLoS ONE*, *10*(9), e0132562.

Elleman, L. G., Condon, D. M., Russin, S. E., & Revelle, W. (2018). The personality of U.S. states: Stability from 1999 to 2015. *Journal of Research in Personality, 72*, 64–72. https://doi.org/10.1016/j.jrp.2016.06.022

Furnham, A., & Cheng, H. (2016). Childhood intelligence predicts adult trait openness: Psychological and demographic indicators. *Journal of Individual Differences, 37*(2), 105–111. https://doi.org/10.1027/1614-0001/a000194

Hill, P. L., & Roberts, B. W. (2011). The role of adherence in the relationship between conscientiousness and perceived health. *Health Psychology*, *30*(6), 797. https://doi.org/10.1037/a0023860

Holm, S. (1979). A simple sequentially rejective multiple test procedure. *Scandinavian Journal of Statistics*, 65-70. https://www.jstor.org/stable/4615733

Kalish, H., Klumpp-Thomas, C., Hunsberger, S., Baus, H. A., Fay, M. P., Siripong, N., … & Sadtler, K. (2021). Undiagnosed SARS-CoV-2 seropositivity during the first 6 months of the COVID-19 pandemic in the United States. *Science Translational Medicine, 13*(601), eabh3826. https://doi.org/10.1126/scitranslmed.abh3826

Klumpp-Thomas, C., Kalish, H., Drew, M., Hunsberger, S., Snead, K., Fay, M. P., ... & Sadtler, K. (2021). Standardization of ELISA protocols for serosurveys of the SARS-CoV-2 pandemic using clinical and at-home blood sampling. *Nature communications*, *12*(1), 113. https://doi.org/10.1038/s41467-020-20383-x

Lin, F. Y., & Wang, C. H. (2020). Personality and individual attitudes toward vaccination: A nationally representative survey in the United States. *BMC Public Health, 20*(1), 1759. <https://doi.org/10.1186/s12889-020-09840-w>

Willroth, E. C., & Atherton, O. E. (2024). Best laid plans: A guide to reporting preregistration deviations. *Advances in Methods and Practices in Psychological Science*, *7*(1), 25152459231213802.
